# Investigation of inert gas washout methods in a new numerical model based on an electrical analogy

**DOI:** 10.1007/s11517-024-03200-1

**Published:** 2024-10-07

**Authors:** Christoph Schmidt, Wasilios Hatziklitiu, Frederik Trinkmann, Giorgio Cattaneo, Johannes Port

**Affiliations:** 1 Institute of Biomedical Engineering, University of Stuttgart, Seidenstraße 36, 70174 Stuttgart, Germany; 2 Pneumology and Critical Care Medicine, Thoraxklinik at University Hospital Heidelberg, Translational Lung Research Center Heidelberg (TLRC), Member of German Center for Lung Research (DZL), Heidelberg, Germany; 3 Department of Biomedical Informatics, Center for Preventive Medicine and Digital Health Baden-Württemberg (CPD-BW), University Medical Center Mannheim, Heidelberg University, Heidelberg, Germany

**Keywords:** Lung model, Compartment model, Electrical analogy, Diffusion, Convection, Multibreath washout

## Abstract

Inert gas washout methods have been shown to detect pathological changes in the small airways that occur in the early stages of obstructive lung diseases such as asthma and COPD. Numerical lung models support the analysis of characteristic washout curves, but are limited in their ability to simulate the complexity of lung anatomy over an appropriate time period. Therefore, the interpretation of patient-specific washout data remains a challenge. A new numerical lung model is presented in which electrical components describe the anatomical and physiological characteristics of the lung as well as gas-specific properties. To verify that the model is able to reproduce characteristic washout curves, the phase 3 slopes (S_3_) of helium washouts are simulated using simple asymmetric lung anatomies consisting of two parallel connected lung units with volume ratios of $\frac{1.25}{\mathrm{0.75}}$, $\frac{1.50}{\mathrm{0.50}}$, and $\frac{1.75}{\mathrm{0.25}}$ and a total volume flow of 250 ml/s which are evaluated for asymmetries in both the convection- and diffusion-dominated zone of the lung. The results show that the model is able to reproduce the S_3_ for helium and thus the processes underlying the washout methods, so that electrical components can be used to model these methods. This approach could form the basis of a hardware-based real-time simulator.

**Graphical abstract:**

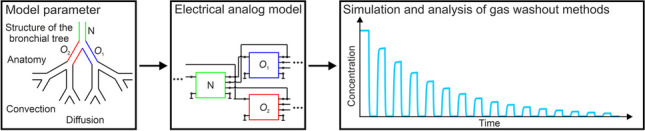

## Introduction

### Medical background

Obstructive pulmonary diseases such as asthma and chronic obstructive pulmonary disease (COPD) are caused by multifactorial processes. Amongst these, respiratory tract inflammation is triggered by allergic reactions [1–4], environmental pollution [5–7], or smoking [8, 9]. These inflammatory processes lead to mucous membrane thickening [10], which narrows the airways [10] and restricts the airflow [11]. This airflow limitation can be determined using established lung function tests such as spirometry [12–14]. For the assessment of the small airways, however, such methods are less suitable [15, 16]. Techniques such as inert gas washout or oscillometry have shown promising results in asthma [17–19] and COPD [20] as well as cystic fibrosis (CF) [21] so they are considered to be more sensitive to these early changes [22].

Inert gas washout methods are divided into single-breath (SBW) and multibreath (MBW) methods. In both variants, different tracer gases can be used. Endogenous nitrogen (N_2_) can either be washed out or exogeneous gases (helium He, sulfur hexafluoride SF_6_, etc.) are washed in while tracer gas concentrations during expiration are recorded. The tracer gases differ in their diffusion properties and are distributed differently in the lung.

The inhomogeneous distribution of gases in the lung, known as ventilation inhomogeneities (VI), is caused on the one hand by lung regions that differ in their mechanical properties and are therefore ventilated differently [23]. These are defined as convection-dependent inhomogeneities (CDI) [24].

On the other hand, the VI are caused by the interplay of convective transport, anatomical, and pathological properties of the lung as well as gas-specific properties (diffusion coefficient). These so-called diffusion-convection interaction-dependent inhomogeneities (DCDI) act in the peripheral airways [25].

Frequently used parameters include lung clearance index (LCI) [26] with different stopping concentrations. This parameter reflects the global VI [27]. Another important parameter is the phase 3 slope (S_3_) [23, 28], which may be important in measuring regional VI from CDI and DCDI [29]. However, the analysis and interpretation of the S_3_ is difficult [29, 30] and requires a better understanding of the processes leading to the CDI and DCDI.

### Numerical models

Computer models and algorithms have a wide range of applications in the field of lung diagnosis and therapy. Machine learning or deep-learning algorithms can help to analyze different datasets [31] including X-ray and CT data [32, 33]. Computational fluid dynamics (CFD) models are used to simulate drug transport [34], for instance, but 3D CFD models in particular can be very time-consuming [24], making them unsuitable for analyzing washout methods.

In addition, numerical models are used to analyze lung mechanics [35–38] and to simulate gas washout methods, as they support the understanding of the fundamental processes of VI and thus potentially the interpretation of experimental or clinical data [24, 39, 40]. However, the general disadvantage of these numerical models is that the simulation time increases with increasing complexity, so simplified model geometries are used to reduce the computational effort and thus focus the VI analysis on specific lung regions such as the acinus [41] or parameters.

Swan et al. [37] developed a model to simulate lung mechanics, which simulates the distribution of ventilation. The conducting airways are modelled by a branched airway network, the ends of which are connected by acini and are considered elastic units. However, the branching structure within the acini and the diffusive gas transport were not taken into account. Therefore, the DCDI cannot be reproduced.

The lung model of Ismail et al. [38] also models only lung mechanics. In contrast to Swan et al. [37], the mechanical parameters such as flow resistance, compliance, and inertia are modelled using electrical variables in order to reduce the computational effort. However, this approach also does not take diffusion into account, which makes this model unsuitable for simulating the DCDI.

Hasler et al. [40] focused on the study of conducting airways. The authors simulated MBW in the human lung by modelling the first 8 to 12 airway generations of the tracheobronchial tree through an asymmetrically dichotomously branched network of tubes with trumpet-shaped lung units at the endpoints. Using this model, the authors were able to investigate the influence of heterogeneities in compliance, residual volume of the airways, and flow resistances and concluded that changes in residual volume and compliance have the greatest effect on the washout curves. However, due to the trumpet-shaped lung units, the diffusive interactions within the acini could not be taken into account in order to keep the computational effort low.

Abbasi and Bozorgmehry Boozarjomehry [24] presented a model that took into account both conductive and respiratory airway asymmetries. The authors were able to generate realistic phase 3 slopes that correlated with measured data. To account for heterogeneities in the convective zone, they modified the distribution of flow resistances based on a symmetrical model geometry, while for the respiratory zone, the exhaled concentration curves were estimated by a function. Although this approach reduces the computational effort, it does not take into account anatomical asymmetries in the convection zone as well as the dynamic interactions at the boundary between the convection and diffusion zones.

High-performance computers can be used to simulate gas transport in more complex numerical models to keep the computational effort to an acceptable level [39], but even here the degree of complexity is limited. In contrast, electrical circuits have the advantage that they can not only be solved numerically, but can in principle be built with electrical components, which could potentially lead to the development of a hardware module that allows real-time simulation regardless of complexity. This would allow the development of patient-specific lung models that could be used to analyze and interpret the measured washout data and thus diagnose obstructive lung disease. Yehya [42] used such electrical components to simulate the diffusive and convective transport of O_2_ and carbon dioxide (CO_2_) in his numerical lung model. However, this approach has not yet been applied to the simulation of washout methods.

In this study, a novel lung model is presented based on electrical components and demonstrates its ability to model transport mechanisms underlying washout methods. The core of the model is a basic computational node in which the geometric, physiological, and gas-specific properties as well as gas transport processes in the respiratory tract are described using electrical components such as resistors, capacitors, and voltage-controlled current sources. By wiring several of these nodes, a complex, branched airway network can be generated. The model was first solved numerically to determine feasibility. In the model test performed here, MBWs of He are simulated with both symmetrical and asymmetrical model geometries in order to check whether the model is able to reproduce characteristic washout curves caused by DCDI. The results under symmetrical conditions are used as a reference.

## Material and methods

The following methodology is used in this work. First, the morphometric data of Weibel’s anatomical lung model [43, p. 136], whose measured lengths (*l*) and diameters (*d*) refer to a lung volume of 4800 ml, are scaled to the respiratory resting position of 3000 ml in adults. Next, a simplified symmetric lung structure is created based on the Weibel model [43] to keep the computational effort acceptable. Then, the symmetrical model is transformed into an asymmetrical model. Subsequently, the gas transport processes are implemented followed by the simulation and evaluation of the washout data.

### Scaling of bronchial volumes

To scale the bronchial volumes, the total volume of Weibel’s lung model (*V*_L,W_) is first calculated from the sum of the volumes of the cylindrical airways and the spherical alveoli (Eq. 1).

1 $${V}_{\text{L,W}}=\sum\limits_{i=0}^{23}{2}^{i}\cdot \left[\frac{\pi }{4}\cdot {d}^{2}\left(i\right)\cdot l\left(i\right)+{m}_{\text{alv,air}}\left(i\right)\cdot \frac{4}{3}\cdot \pi \cdot \frac{{d}_{\text{alv}^{3}}}{8}\right]$$

In the formula, *m*_alv,air_(*z*) is the number of alveoli per airway in a generation *z* and *d*_alv_ is the average diameter of an alveolus. Weibel gives *d*_alv_ as a function of the total lung volume (*V*_L_) according to Eq. 2 [43, p. 66], where the factor $1.54\cdot {10}^{-3}$ was determined empirically. For *V*_L_ = 3000 ml, as it is used here, *d*_alv_ is 222 μm.

2 $${d}_{\text{alv}}=1.54\cdot {10}^{-3}\cdot {V}_{\text{L}^{1/3}}$$

With the parameters *d*, *l*, and *m*_alv_ given by Weibel [43] and *d*_alv_ of 222 μm, *V*_L,W_ = 3368 ml results from Eq. 1. In order to correct the difference of 368 ml, the parameters *d* and *l* of the airways in generations 4 to 23 are each multiplied by the scaling factor *s* and the equation for the scaled lung volume of the Weibel model (${\widetilde{\mathrm{V}}}_{{\mathrm{L}}\mathrm{,}{\mathrm{W}}}$) is set up (Eq. 3). The parameters *m*_alv,air_ and *d*_alv_ remain unchanged. The airways of generations 0 to 3 are not scaled as explained in Section 2.2.1.

3 $${\widetilde V}_\text{L,W}=\sum_{i=0}^32^i\cdot\frac{\pi}{4}\cdot d^2\left(i\right)\cdot l\left(i\right)+\sum_{j=4}^{23}2^j\cdot\left[\frac{\pi}{4}\left(s\cdot d\left(j\right)\right)^2\cdot s\cdot l\left(j\right)+m_{\text{alv,air}}\left(j\right)\cdot\frac{4}{3}\cdot\pi\cdot\frac{d_{\text{alv}^3}}{8}\right]$$

With approach ${\mathrm{V}}_{{\mathrm{L}}\mathrm{,}{\mathrm{W}}}-{\widetilde{\mathrm{V}}}_{{\mathrm{L}}\mathrm{,}{\mathrm{W}}} \, \mathrm{=} \, \mathrm{368 }{\mathrm{ml}}$ (Eq. 1, Eq. 3), the scaling factor *s* can be calculated with Eq. 4 and is: *s* = 0.917.

4 $$s={\left(1-\frac{368 \text{ ml}}{\frac{\pi }{4}\cdot \sum_{i=4}^{23}{2}^{i}\cdot {d}^{2}\left(i\right)\cdot l\left(i\right)}\right)}^{1/3}$$

### Creation of a simplified symmetrical lung model

#### Structure of the lung model

**Fig. 1:**
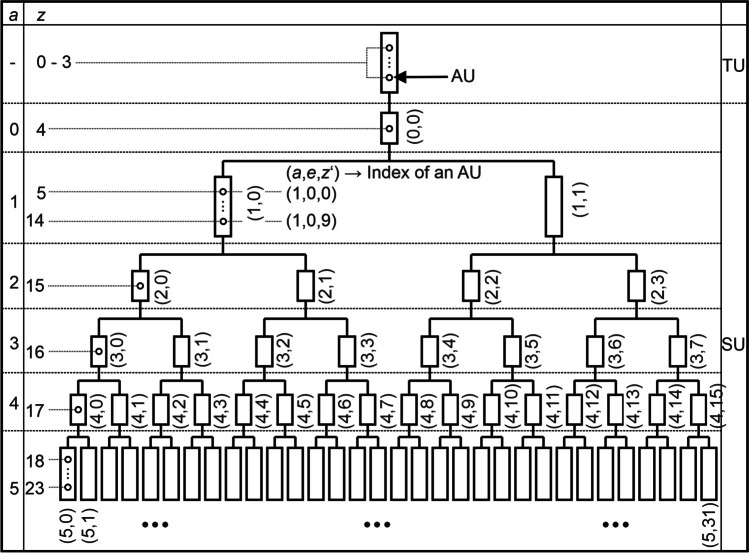
Schematic representation of the lung model. The elements in the segment unit are indexed via the tuple (*a*, *e*), where *a* and *e* stand for the area and element according to the scheme Henry et al. used [44]. The circles represent the airway units. For reasons of clarity, they are only displayed in the first element of a panel. More information in the text

The lung model in this work is divided into 2 parts (Fig. 1). The first part includes the airways of generations 0 to 3, which approximately correspond to the trachea, main bronchi, lobar bronchi, and segment bronchi [43, p. 125]. This part is called as trachea unit (TU). The second part, which includes generations 4 to 23, is defined as segment unit (SU). This structure was chosen since in subsequent works, heterogeneities at the segment level are to be examined by connecting several lung segment units in parallel. For this reason, the airways in the TU are assumed to be symmetrical and only the lengths and diameters from generation 4 onwards were scaled (Eq. 3), to consider the heterogeneities at segment level in particular. The results obtained here can thus be compared with those of future  work.

In addition, so-called airway units (AU) are used, in which the incoming and outgoing gas volume flows are balanced. They are shown as circles in Fig. 1.

#### Trachea unit

In this paper, the TU is modeled as a tube with 10 anatomically identical AUs connected in series (Fig. 1), whose volume *V*_AU-TU_ can be calculated as one-tenth of the total volume of the generations 0 to 3:

5 $${V}_{\text{AU-TU}}=\frac{1}{10}\cdot \sum_{i=0}^{3}{2}^{i}\cdot \frac{\pi }{4}\cdot {d}^{2}\left(i\right)\cdot l\left(i\right)$$

Although the TU represents generations 0 to 3, 10 AUs are chosen instead of 4. This is due to the observation that fewer than 10 AUs lead to increasing numerical smearing.

#### Segment unit

The SU corresponds to a branched pipe network and is divided into 6 areas, including the entrance in area 0, which makes it possible to investigate the effects of geometric asymmetries in the convection-dominated zone in area 1 as well as at different positions within the diffusion-dominated zone between its beginning in area 2 and the beginning of the respiratory zone in area 5. The transition between these two zones (Table 1) results from the increase in cross section due to the increasing branching of the bronchi, which causes the flow rate to decrease and can be calculated using the Peclet number (Pe), which is greater than 1 in the case of dominant convection and less than 1 in the case of dominant diffusion. The calculation of Pe is explained in more detail in Section 2.4.2.

**Table 1:** Peclet numbers and diffusional resistances for He-N_2_ with *D* = 0.6 cm^2^/s along the lung axis *x* and the parameters *l*_*N*_, *A*_*N*_, and ${\widehat{\mathrm{Q}}}_{{N}\mathrm{,in}}$ of the *N*’th AU needed to calculate Pe_*N*_ (Eq. 26) and *R*_D,*N→M*_ (Eq. 29). The grey marking represents the transition between the convection- and diffusion-dominated zone of He-N_2_. For further information, see Section 2.4.

AE	*z*	*l*_*N*_ (cm)	Axis *x* (cm)	*A*_*N*_ (cm^2^)	${\widehat{\mathrm{Q}}}_{{\mathrm{N}}\mathrm{,in}}$ (cm^3^/s)	Pe_*N*,in_ (*D*)	*R*_D,*N→M*_ (s/cm^3^)
TU	0–3	1.945	0	2.435	250.000	332.780	10.00E+100
			1.945–17.50				1.33E+00
SU-(0,0)	4	1.161	19.06	2.094	250.000	309.074	1.24E+00
SU-(1,0)	5	0.980	20.13	1.304	125.000	171.045	1.37E+00
	6	0.827	21.03	1.665	125.000	112.990	9.04E−01
	7	0.697	21.79	2.181	125.000	72.800	5.82E−01
	8	0.588	22.43	2.928	125.000	45.749	3.66E−01
	9	0.496	22.98	4.030	125.000	28.041	2.24E−01
	10	0.419	23.43	5.687	125.000	16.763	1.34E−01
	11	0.353	23.82	8.229	125.000	9.774	7.82E−02
	12	0.298	24.15	12.207	125.000	5.559	4.45E−02
	13	0.251	24.42	18.567	125.000	3.083	2.47E−02
	14	0.212	24.65	28.953	125.000	1.668	1.33E−02
SU-(2,0)	15	0.179	24.85	23.145	62.500	0.880	1.41E−02
SU-(3,0)	16	0.151	25.01	18.970	31.250	0.453	1.45E−02
SU-(4,0)	17	0.127	25.15	15.941	15.625	0.227	1.46E−02
SU-(5,0)	18	0.107	25.27	13.735	7.795	0.111	1.43E−02
	19	0.091	25.37	24.265	7.740	0.053	6.81E−03
	20	0.077	25.45	43.952	7.576	0.024	3.17E−03
	21	0.065	25.52	81.624	7.027	0.010	1.44E−03
	22	0.054	25.58	155.416	5.929	0.004	6.38E−04
	23	0.046	25.63	303.400	3.733	0.001	2.76E−04

Due to the branched structure of the SU, each area consists of 2^*a*^ (0 ≤ *a* ≤ 5) elements, which are referred to as surface elements (AE). Each AE within an area has the same number of AUs (*m*_AU_(*a*); Fig. 1 circles). In contrast to the TU, an AU within the SU represents a generation.

Each AU within an area *a* (0 ≤ *a* ≤ 5) and element *e* (0 ≤ *e* ≤ 2^*a*^-1) is indexed via the triple (*a*,*e*,*z*'), where *z*′ indicates the position within an AE (0 ≤ z′ ≤ *m*_AU_(*a*)-1), as shown in Fig. 1. This indexing is important for the later transformation into the asymmetric model. The first two indices *a* and *e* were assigned according to the scheme Henry et al. used [44]. With the function *φ*(*a*,*z*′) (Eq. 6), the corresponding “global” airway generation *z* can be calculated for each *a* and *z*′.

6 $$\varphi \left(a,{z}^{\prime}\right)=z=\left\{\begin{array}{c}{z}^{\prime}+4, a=0\\ {z}^{\prime}+4+\sum\nolimits_{i=0}^{a-1}{m}_\text{AU}\left(i\right), a>0\end{array}\right.$$

As a result of the structure of the SU, each AU represents to a group of 2^*φ*(*a*,*z*‘)-*a*^ anatomically identical airways of the Weibel model. The volumes of the AU in the symmetrical model (*V*_AU-SU,sym_(*a*,*e*,*z*‘)) are identical for all *e* (Eq. 7) and can be calculated from the “standard volume” *V*_AU-SU_(*a*,*z*‘) according to Eq. 8.

7 $${V}_\text{AU,SU, sym}\left(a,e,{z}^{\prime}\right)={V}_\text{AU,SU}\left(a,{z}^{\prime}\right), \forall e\in \left\{0\le e\le {2}^{a}-1\right\}$$

8 $${V}_\text{AU,SU}\left(a,{z}^{\prime}\right)={2}^{\varphi \left(a,{z}^{\prime}\right)-a}\cdot \left[{s}^{3}\cdot \frac{\pi }{4}\cdot {d}^{2}\left(\varphi \left(a,{z}{\prime}\right)\right)\cdot l(\varphi(a,z^\prime))+{m}_\text{alv, air}\left(\varphi \left(a,{z}^{\prime}\right)\right)\cdot \frac{4}{3}\cdot \pi \cdot \frac{{d}_\text{alv}^{3}}{8}\right]$$

The total volume of the symmetrical lung model (*V*_L,sym_) results from the sum of the volumes of the AUs of TU (Eq. 5) and SU (Eq. 7, Eq. 8) and can be calculated with Eq. 9.

9 $${V}_\text{L,sym}=10\cdot {V}_\text{AU-TU}+\sum\limits_{a=0}^{5}\sum\limits_{{z}^{\prime}=0}^{{m}_\text{AU}\left(a\right)-1}{2}^{a}\cdot {V}_\text{AU-SU}\left(a,{z}^{\prime}\right)$$

The AUs in the TU and in areas 0 to 3 of the SU are not covered with alveoli and their volume *V*_Dead_ of 144.6 ml is called dead space.

### Transformation of the symmetric model into an asymmetric model

The following 3 conditions apply to the transformation from the symmetric to the asymmetric model:

- Condition 1: The total lung volumes of the symmetric and asymmetric models are identical (*V*_L,sym_ = *V*_L,asym_).
- Condition 2: The volume of the TU is identical to the total volume within the generations 0 to 3 of the symmetrical Weibel model.
- Condition 3: For the transformation, the volumes of the AUs (*a*,*e*,*z*′) of the symmetrical model are factorized. For this purpose, a volume factor *f* (*a*,*e*) is defined for each *e'*th AE within *a*, which is multiplied with the volumes of all AUs in the *e*th AE of the symmetrical model. Each factor is inherited from a parent area to the subsequent daughter areas. This means, all the volumes of the AUs of the subsequent AEs must be multiplied with this factor. Thus, the volume of an AU (*a*,*e*,*z*′) in the asymmetric model ${V}_{{\mathrm{AU}}-{\mathrm{SU}}\mathrm{,a}{\mathrm{sym}}}\mathrm{(}{a}\mathrm{,}{e}\mathrm{,}{z}^{\prime}\mathrm{)}$ (Eq. 10) results from the volume ${V}_{{\mathrm{AU}}-{\mathrm{SU}}}\left({a}\mathrm{,}{z}^{\prime}\right)$ of the symmetric model and the product of both its own factor *f*(*a*,*e*) and the factors ${f}\mathrm{(}{a}-{i}\mathrm{,}{e} \, {\mathrm{div}} \, {2}^{i}\mathrm{)}$ of all its predecessor elements (1 ≤ *i* ≤ *a*). The div operator gives the integer result of a division.
  10 $$V_\text{AU,SU,asym}\left(a,e,z^{'}\right)=\prod \limits_{i=0}^af\left(a-i,\;e\;\text{div}\;2^i\right)\cdot V_\text{AU,SU}\left(a,z^{'}\right)$$

With conditions 2 and 3 including Eq. 10, the lung volume of the asymmetric model (*V*_L,asym_) can be specified according to Eq. 11.

11 $$V_\text{L,asym} = 10\cdot V_\text{AU-TU}+\sum\limits_{a=0}^5\sum\limits_{z'=0}^{m_\text{AU}\left(a\right)-1}\sum\limits_{e=0}^{2^a-1}\prod\limits_{i=0}^af\left(a-i,\;e\;\text{div}\;2^i\right)\cdot V_\text{AU-SU}\left(a,z'\right)$$

In order to fulfil condition 1 (*V*_L,sym_ = *V*_L,asym_; Eq. 9, Eq. 11), the following condition must be fulfilled:

12 $$\sum\limits_{e=0}^{{2}^{a}-1}\prod \limits_{i=0}^{a}f\left(a-i,\;e\;\text{div}\;{2}^{i}\right)={2}^{a}$$

This condition is fulfilled, if *f*(*a*,*e*) is:

13 $$f\left(a,e\right)+f\left(a,e+1\right)=2, for e=\mathrm{0,2},4,\dots ,{2}^{a}-1, f\left(a,e\right)>0$$

#### Lengths, diameters and number of alveoli

The diameters (*d*_asym_(*a*,*e*,*z′*), Eq. 14), lengths (*l*_asym_(*a*,*e*,*z′*), Eq. 15), and number of alveoli (*m*_alv,asym_(*a*,*e*,*z′*), Eq. 16) of the AUs in the asymmetric model can be calculated form Eq. 8, if the asymmetric volumes from Eq. 10 are used instead of the symmetric ones. The cross-sectional area (*A*_asym_(*a*,*e*,*z′*), Eq. 17) of the AUs can be calculated from their diameters.

14 $${d}_\text{asym}\left(a,e,{z}^{\prime}\right)={\left({2}^{\varphi \left(a,{z}^{\prime}\right)-a}\right)}^\frac{1}{2}\cdot s\cdot \prod \limits_{i=0}^{a}{f}^{{~}^{1}\!\left/ \!{~}_{3}\right.}\left(a-i,\;e\;\text{div}\;{2}^{i}\right)\cdot d\left(\varphi \left(a,{z}^{\prime}\right)\right)$$

15 $${l}_\text{asym}\left(a,e,{z}^{\prime}\right)=s\cdot \prod \limits_{i=0}^{a}{f}^{{~}^{1}\!\left/ \!{~}_{3}\right.}\left(a-i,\;e\;\text{div}\;{2}^{i}\right)\cdot l\left(\varphi \left(a,{z}^{\prime}\right)\right)$$

16 $${m}_\text{asym}\left(a,e,{z}^{\prime}\right)={2}^{\varphi \left(a,{z}^{\prime}\right)-a}\cdot \prod \limits_{i=0}^{a}f\left(a-i,\;e\;\text{div}\;{2}^{i}\right)\cdot {m}_\text{alv,air}\left(\varphi \left(a,{z}^{\prime}\right)\right)$$

17 $${A}_\text{asym}\left(a,e,{z}^{\prime}\right)=\frac{\pi }{4}\cdot {d}_\text{asym}^{2}\left(a,e,{z}^{\prime}\right)$$

#### Volume flows

The magnitude of the time-dependent total volume flow in TU (*Q*_TU_(*t*)), which is a rectangular function (Section 2.4.2), of 250 ml/s is assumed with a tidal volume of 500 ml and a respiratory rate of 15 1/min. This flow is generated by the change in volume of the individual alveoli over time $\left(\frac{{\mathrm{dV}}_{\mathrm{alv}}\mathrm{(t)}}{\mathrm{dt}}\right)$ during respiration. The change in volume of an alveolus results from the volume flow in the alveolus (*Q*_alv_(t)) and can be calculated according to Eq. 18 from *Q*_TU_(t) and the sum of all alveoli for the symmetrical model.

18 $${Q}_\text{alv}\left(t\right)=\frac{{Q}_\text{TU}\left(t\right)}{\sum\nolimits_{z=0}^{23}{2}^{z}\cdot {m}_\text{alv/air}\left(z\right)}$$

By factorizing the number of alveoli (Eq. 16) as a consequence of the asymmetrical model, the volume flows of the alveoli in the AUs are factorized accordingly (Eq. 19). Therefore, volume and volume flow of an AU are proportional to each other. The volume flow at the entrance of an AU *Q*_AU-in_(*a*,*e*,*z*‘) results from the flow in its alveolar volume and the flow in the alveolar volumes of all subsequent AUs (Eq. 20).

19 $${Q}_\text{AU-alv}\left(t,a,e,{z}^{\prime}\right)={m}_\text{alv, asym}\left(a,e,{z}^{\prime}\right)\cdot {\mathrm{Q}}_{\mathrm{alv}}\left(t,\varphi \left(a,{z}^{\prime}\right)\right)$$

20 $${Q}_\text{AU-in}\left(t,a,e,{z}^{\prime}\right)=\sum\limits_{i=0}^{5-a}\sum\limits_{j=0}^{{2}^{i}-1}\sum\limits_{k=0+x\left(i\right)\cdot {z}^{\prime}}^{{m}_\text{AU}\left(a+i\right)-1}{Q}_\text{AU-alv}\left(t, a+i,e\cdot {2}^{i}+j,k\right);\text{ with }\;x\left(i\right)=\left\{\begin{array}{c}1,\;\text{ for }\;i=0\\ 0, \text{ else}\end{array}\right.$$

The indexing of the AUs is important for describing the anatomical structure of the lung model. In the following sections, however, *N* is used for indexing an individual AU for the sake of simplification.

### Implementation of the gas transport processes

For the estimation of the tracer gas volume over time $\frac{d}{dt}\left({V}_{N}\left(t\right)\cdot {\chi }_\text{Tr,N}\left(t\right)\right)$ in the *N*-th AU, the finite difference method is used. For this purpose, the inflowing and outflowing tracer gas volume flows ${\dot{\mathrm{V}}}_{\mathrm{Tr},N,\mathrm{in}}\left({t}\right)$ and ${\dot{\mathrm{V}}}_{\mathrm{Tr,}{N}\mathrm{,out}}\left({t}\right)$ are determined under the assumption that the gas transport only takes place through one-dimensional diffusion and convection.

21 $$\frac{\text{d}}{\textrm{dt}}\left({V}_{N}\left(t\right)\cdot {\chi }_{\text{Tr},N}\left(t\right)\right) = {\dot{V}}_{\text{Tr},N,\text{in}}\left(t\right)-{\dot{V}}_{\text{Tr},N,\text{out}}\left(t\right)$$

22 $${\dot{V}}_{\text{Tr},N,\text{in}}\left(t\right)=\frac{{Q}_{N,\text{in}}{\left(t\right)}+\left|{Q}_{N,\text{in}}{\left(t\right)}\right|}{2}\cdot {\chi }_{\text{Tr},M}\left(t\right)+\sum\limits_{\forall i}\frac{-{Q}_{{O}_{i},\text{in}}{\left(t\right)}+\left|{Q}_{{O}_{i},\text{in}}{\left(t\right)}\right|}{2}\cdot {\chi }_{\text{Tr},{O}_{i}}\left(t\right)+\frac{-D\cdot {A}_{N}}{\frac{1}{2}\cdot \left({l}_{N}+{l}_{M}\right)}\cdot \left({\chi }_{\text{Tr},N}\left(t\right)-{\chi }_{\text{Tr},M}\left(t\right)\right)$$

23 $${\dot{V}}_{\text{Tr},N,\text{out}}\left(t\right)=\frac{-{Q}_{N,\text{in}}{\left(t\right)}+\left|{Q}_{N,\text{in}}{\left(t\right)}\right|}{2}\cdot {\chi }_{\text{Tr},N}\left(t\right)+\left[\sum_{\forall i}\frac{{Q}_{{O}_{i},\text{in}}{\left(t\right)}+\left|{Q}_{{O}_{i},\text{in}}{\left(t\right)}\right|}{2}\right]\cdot {\chi }_{\text{Tr},N}\left(t\right)+\sum_{\forall i}\frac{-D\cdot {A}_{{O}_{j}}}{\frac{1}{2}\cdot \left({l}_{N}+{l}_{{O}_{j}}\right)}\cdot \left({\chi }_{\text{Tr},{O}_{j}}\left(t\right)-{\chi }_{\text{Tr},N}\left(t\right)\right)$$

#### Diffusion

**Fig. 2:**
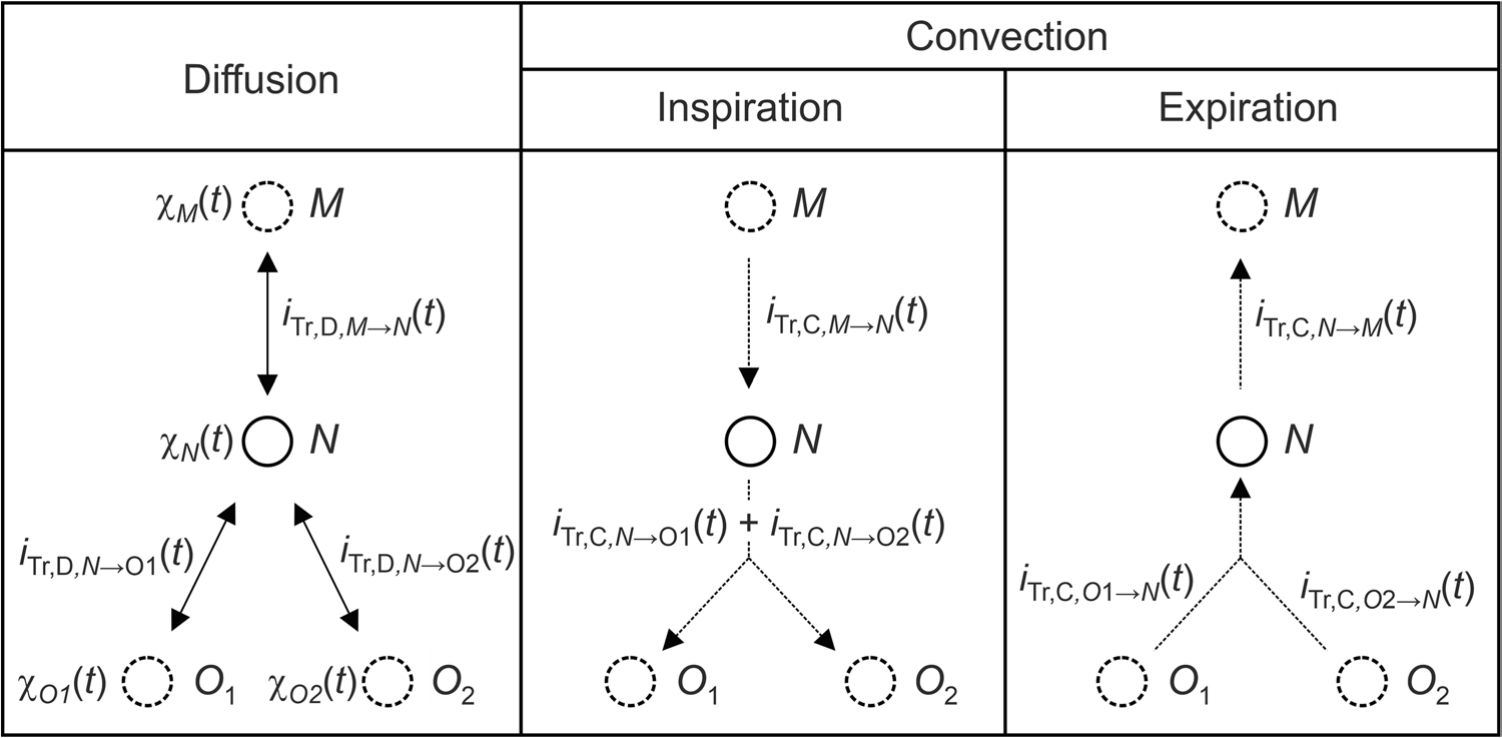
Comparison of non-directional diffusive and directional convective gas transport at a bifurcation

The diffusive gas transport is based on Fick’s first law and is dependent on the concentration gradient of the gas. Here, the calculated concentration is the dimensionless mole fraction $\chi$. It is a non-directional transport process, which means that diffusion can take place in both directions independently of inspiration and expiration, which is shown schematically in Fig. 2. The AUs labeled with *M*, *O*1, and *O*2 are predecessor as well as first and second successors of the *N*’th AU. For ${\dot{V}}_{\mathrm{Tr},N,\mathrm{in}}\left({\mathrm{t}}\right)$ and ${\dot{V}}_{\mathrm{Tr},N,\mathrm{out}}\left({\mathrm{t}}\right)$, the concentration differences between the neighboring AUs *M*-*N* (${\chi }_{\mathrm{Tr},N}\left({\mathrm{t}}\right)-{\chi }_{\mathrm{Tr},M}\left({\mathrm{t}}\right)$), *N*-*O*1 (${\chi }_{\mathrm{Tr},N}\left({\mathrm{t}}\right)-{\chi }_{\mathrm{Tr},O1}\left({\mathrm{t}}\right)$), and *N*-*O*2 (${\chi }_{\mathrm{Tr},N}\left({\mathrm{t}}\right)-{\chi }_{\mathrm{Tr},O2}\left({\mathrm{t}}\right)$) have to be evaluated (Eq. 22, Eq. 23).

The molecular diffusion coefficient (*D*) is valid for binary gas mixtures. In this work, He-N_2_ is used, which has a diffusion coefficient of 0.6 cm^2^/s [41], where N_2_ is the carrier and He the tracer gas. According to their units, the terms $\frac{D\cdot {A}_{N}}{\frac{1}{2}\left({l}_{N}+{l}_{M}\right)}$ (Eq. 22) and $\frac{D\cdot {{A}_{o}}_{j}}{\frac{1}{2}\left({l}_{N}+{{l}_{o}}_{j}\right)}$ (Eq. 23) correspond to diffusive volume flows between two neighboring AUs. The term $\frac{1}{2}\cdot \left({l}_{N}+{l}_{M}\right)$ is the distance between *N* and *M*.

#### Convection

The volume flows are regarded as rectangular functions (Eq. 24).

24 $$Q_{N,\text{AU-in}}\left(t\right)=\left\{\begin{array}{c}\begin{array}{cc}{\widehat Q}_{N,\mathrm{AU-in}},\text{ for }\left(n-1\right)\cdot T\leq t<n\cdot T-\frac{T}{2}:\text{Inspiration}\end{array}\\ \begin{array}{cc}-{\widehat Q}_{N,\text{AU-in}},\text{ for }n\cdot T-\frac{T}{2}\leq t<n\cdot T:\text{ Expiration}\end{array}\end{array}\right.,\;n=1,2,3,...$$

In contrast to diffusion, convective gas transport is dependent on inspiration and expiration and therefore directed (Fig. 2). For this reason, the reversal of the flow direction (Eq. 24) must be taken into account for the balancing. During inspiration, gas flows from *M* to *N* and from *N* to *O*1 or *O*2 (Fig. 2). Expiration occurs in the opposite direction. This reversal of direction can be described mathematically with the term $\frac{\pm {Q}_{N,\text{in}}\left(t\right)+\left|{Q}_{N,\text{in}}\left(t\right)\right|}{2}$ (Eq. 22, Eq. 23), which represents the explicit upwind method [45].

The volume of an AU *V*_*N*_(*t*) (Eq. 25) can be calculated from the non-time-dependent ${V}_{{\mathrm{AU}}-{\mathrm{SU}}\mathrm{,a}\mathrm{sym},N}$ (Eq. 10) and the time-dependent alveolar volume change (Eq. 19).

25 $${V}_{N}\left(t\right)={V}_{{\mathrm{AU}}-{\mathrm{SU,asym,}}N}+{\int }_{0}^{t}{Q}_{\text{AU-alv},N}\left(\tau \right)\cdot d\tau$$

The ratio of ${\widehat{Q}}_{N,\mathrm{in}}$ and $\frac{D\cdot {A}_{N}}{\frac{1}{2}\cdot \left({l}_{N}+{l}_{M}\right)}$ corresponds to the Peclet number [44] at the inlet of *N* (Pe_*N*,in_) Eq. 26, which is used to determine the transition between the convection- and diffusion-dominated zone for He (Table 1; Section 2.2.3).

26 $$\text{Pe}_{N,\mathrm{in}}=\frac{{\widehat{Q}}_{N,\mathrm{in}}\cdot \frac{1}{2}\cdot \left({l}_{N}+{l}_{M}\right)}{D\cdot {A}_{N}}$$

The transit time $\Delta {t}_\text{Dead}$ through the dead space of *V*_Dead_ = 144.6 ml is $\Delta {t}_\text{Dead}$ = 0.6s according to Eq. 27.

27 $$\Delta {t}_\text{Dead}=\frac{{V}_\text{Dead}}{250 \text{ml/s}}=\frac{144.6 \mathrm{ml}}{250 \mathrm{ml/s}}=0.6\mathrm{s}$$

#### Electrical analogy

28 $$\begin{array}{c}\frac{\mathrm{d}}{\mathrm{d}t}\left({V}_{N}\left(t\right)\cdot {\chi }_{\mathrm{Tr},N}\left(t\right)\right)={\dot{V}}_{\mathrm{Tr},N,\mathrm{in}}\left(t\right)-{\dot{V}}_{\mathrm{Tr},N,\mathrm{out}}\left(t\right)\\ \frac{\mathrm{d}}{\mathrm{d}t}\left({C}_{N}\left(t\right)\cdot {u}_{\mathrm{Tr},N}\left(t\right)\right)={i}_{\mathrm{Tr},N,\mathrm{in}}\left(t\right)-{i}_{\mathrm{Tr},N,\mathrm{out}}\left(t\right)\\ {i}_{\mathrm{Tr},N}\left(t\right)={i}_{\mathrm{Tr},N,\mathrm{in}}\left(t\right)-{i}_{\mathrm{Tr},N,\mathrm{out}}\left(t\right)\end{array}$$

29 $$\begin{array}{l}{\dot V}_{\mathrm{Tr},N,\mathrm{in}}\left(t\right)=\frac{Q_{N,\mathrm{in}}\left(t\right)+\left|Q_{N,\mathrm{in}}\left(t\right)\right|}2\cdot\chi_{\mathrm{Tr},M}\left(t\right)+\sum\limits_{\forall j}\frac{-Q_{O_j,\mathrm{in}}\left(t\right)+\left|Q_{O_j,\mathrm{in}}\left(t\right)\right|}2\cdot\chi_{\mathrm{Tr},O_j}\left(t\right)+\frac{-D\cdot A_N}{\frac12\cdot\left(l_N+l_M\right)}\cdot\left(\chi_{\mathrm{Tr},N}\left(t\right)-\chi_{\mathrm{Tr},M}\left(t\right)\right)\\i_{\mathrm{Tr},N,\mathrm{in}}\left(t\right)=\frac1{R_{\mathrm{c},N,\mathrm{in}}\left(t\right)}\cdot u_{\mathrm{Tr},M}\left(t\right)+\sum\limits_{\forall j}\frac1{R_{\mathrm{c},O_j,\mathrm{in}}\left(t-T/2\right)}\cdot u_{Tr,O_j}\left(t\right)+\frac1{R_{\mathrm{D},M\rightarrow N}}\cdot\left(u_{\mathrm{Tr},M}\left(t\right)-u_{\mathrm{Tr},N}\left(t\right)\right)\\i_{\mathrm{Tr},N,\mathrm{in}}\left(t\right)=i_{\mathrm{Tr,c},M\rightarrow N}\left(t\right)+\sum\limits_{\forall j}i_{\mathrm{Tr,c},O_j\rightarrow N}\left(t\right)+i_{\mathrm{Tr,D},M\rightarrow N}\left(t\right)\end{array}$$

30 $$\begin{array}{l}{\dot V}_{\mathrm{Tr},N,\mathrm{out}}\left(t\right)=\frac{-Q_{N,\mathrm{in}}\left(t\right)+\left|Q_{N,\mathrm{in}}\left(t\right)\right|}2\cdot\chi_{\mathrm{Tr},N}\left(t\right)+\left[\sum\limits_{\forall j}\frac{Q_{O_j,\mathrm{in}}\left(t\right)+\left|Q_{O_j,\mathrm{in}}\left(t\right)\right|}2\right]\cdot\chi_{\mathrm{Tr},N}\left(t\right)+\sum\limits_{\forall j}\frac{-D\cdot A_N}{\frac12\cdot\left(l_N+l_M\right)}\cdot\left(\chi_{\mathrm{Tr},O_j}\left(t\right)-\chi_{\mathrm{Tr},N}\left(t\right)\right)\\i_{\mathrm{Tr},N,\mathrm{out}}\left(t\right)=\frac1{R_{\mathrm{c},N,\mathrm{in}}\left(t-T/2\right)}\cdot u_{\mathrm{Tr},N}\left(t\right)+\left[\sum\limits_{\forall j}\frac1{R_{\mathrm{c},O_j,\mathrm{in}}\left(t\right)}\right]\cdot u_{\mathrm{Tr},O_j}\left(t\right)+\sum\limits_{\forall j}\frac1{R_{\mathrm{D},M\rightarrow O_j}}\cdot\left(u_{\mathrm{Tr},N}\left(t\right)-u_{\mathrm{Tr},O_j}\left(t\right)\right)\\i_{\mathrm{Tr},N,\mathrm{out}}\left(t\right)=i_{\mathrm{Tr,c,}N\rightarrow M}\left(t\right)+\sum\limits_{\forall j}i_{\mathrm{Tr,c},N\rightarrow O_j}\left(t\right)+i_{\mathrm{Tr,D},N\rightarrow O_j}\left(t\right)\end{array}$$

For the mechanical quantities of the transport equations for diffusion and convection in Eq. 21, Eq. 22, and Eq. 23, electrical quantities were derived. The comparison between these quantities is shown in Table 2. The diffusive volume flow corresponds to a passive, electrical conductance, which is the reciprocal of an electrical resistance *R*_D_ [42]. The values of the diffusional resistances of the symmetrical model are given in Table 1. The value for the first AU is explained in Section 2.5.2.

**Table 2:** Comparison of the electrical and mechanical quantities

Electrical quantity	Mechanical quantity	
	Diffusion	Convection
Electrical resistance *R* in [Ω]	Diffusional resistance ${R}_\text{D}=\frac{\Delta l}{D\cdot A} \text{ in } \left[\frac{\mathrm{s}}{{\mathrm{m}}^{3}}\right]$	Convectional resistance ${R}_\text{C}=\frac{1}{Q} \text{in} \left[\frac{\text{s}}{{\mathrm{m}}^{3}}\right]$
Electrical current *i* in [A]	Volume flow of the tracer gas ${\dot{V}}_{\mathrm{Tr}}\text{ in }\left[\frac{{\mathrm{m}}^{3}}{\mathrm{s}}\right]$	
Electrical voltage *u* in [V]	Molar fraction (concentration) of the tracer gas to the gas mixture *χ*_Tr_ dimensionless	
Electrical capacity *C* in [F]	Volume of an AU *V* in[(m^3^]	

**Fig. 3:**
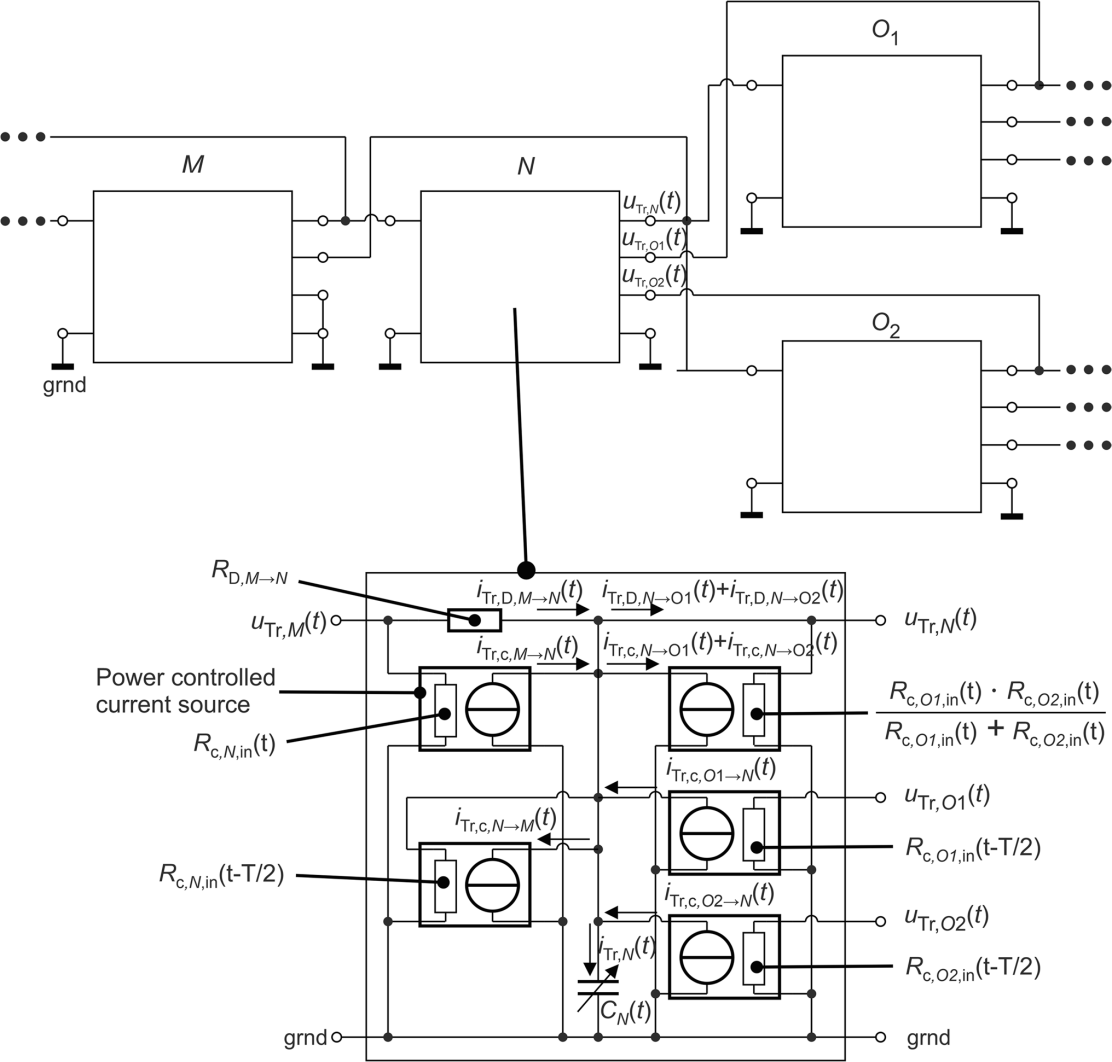
Electrotechnical representation using the example of the bifurcation from Fig. 2. See text for details

The convective volume flow through an AU is modeled with voltage-controlled current sources [42] and electrical resistances (Fig. 3)_._ Since convection is a directional transport process, in total five current sources are needed for each AU and 3 for the incoming and 2 for the outgoing volume flows (Fig. 2, Fig. 3). The electrical voltages (*u*), currents (*i*), and capacities (*C*) correspond to the molar fractions (*χ*), tracer gas volume flows (${\dot{V}}_{\mathrm{Tr}}$), and the volumes (*V*). The transport equations Eq. 21, Eq. 22, and Eq. 23 are implemented by electrically connecting the AUs on the basis of the electrical analogies, as is shown schematically in Fig. 3 using the example of the bifurcation from Fig. 2. The model was implemented in MATLAB^®^ Simulink^®^ using the Simscape^®^ Toolbox.

The lung model developed by Yehya [42] is also based on electrical components and can be used to simulate the convective and diffusive transport of O_2_ and CO_2_ in the airways, alveoli, and blood capillaries taking into account the gas exchange through the alveolar-capillary membrane and lung mechanics. For this purpose, 4 special basic calculation nodes were used, all modelled with electrical circuits, and form the basis for a branched compartment model of the lung. The volumes of the airways and capillaries are constant over time; those of the alveoli are time-dependent. In contrast to Yehya [42], the pulmonary mechanics in this work are not modelled with a separate circuit, since the volume flows of all alveoli have the same phase and are described by rectangular functions (Eq. 24). Since the washout methods use gases that are considered inert and therefore do not diffuse through the alveolar-capillary membrane, the capillaries are not modelled in this work and the compliance and volumes of the alveoli are considered in one computational node, thereby reducing the complexity of the model and thus the simulation time.

#### Time constants

The diffusive and convective time constants ${\tau }_{\mathrm{D,}N}(t)$ and ${\tau }_{\mathrm{c,}N}(t)$ of the system based on Eq. 28, Eq. 29, and Eq. 30 are obtained from the products of the electrical resistors *R*_D_ and *R*_c_ with the respective capacitances *C* (Eq. 31, Eq. 32).

31 $${\tau }_{\text{D},N}\left(t\right)={R}_{\text{D},N}\cdot {C}_{N}\left(t\right)=\frac{\frac{1}{2}\cdot \left({l}_{N}+{l}_{M/O1/O2}\right)}{D\cdot {A}_{N}}\cdot {V}_{N}\left(t\right)$$

32 $${\tau }_{\text{c},N}\left(t\right)={R}_{\text{c},N}\left(t\right)\cdot {C}_{N}\left(t\right)=\frac{1}{{Q}_{N/O1/O2}}\cdot {V}_{N}\left(t\right)$$

Assuming that these time constants and their associated parameters correspond to the symmetric model, the time constants of the asymmetric models ${\tau }_{\mathrm{D,}N}^{\prime}$ and ${\tau }_{\mathrm{c,}N}^{\prime}$ can be determined (Eq. 33, Eq. 34) by replacing the parameters *V*, *l*, *A*, and *Q* with their factorized values from equations Eqs. 10, 15, 17, 16 and 19 with *f*_tot_ as the product of all factors *f*(*a*,*e*).

33 $${\tau }_{\text{D},N}^{\prime}\left(t\right)=\frac{\frac{1}{2}\cdot \left({f}_{\text{tot},N}^{{~}^{1}\!\left/ \!{~}_{3}\right.}\cdot {l}_{N}+{f}_{\text{tot}, N/O1/O2}^{{~}^{1}\!\left/ \!{~}_{3}\right.}\cdot {l}_{M/O1/O2}\right)}{D\cdot {f}_{\text{tot},N}^{{~}^{2}\!\left/ \!{~}_{3}\right.}\cdot {A}_{N}}\cdot {f}_{\text{tot},N}\cdot {V}_{n}\left(t\right)=\frac{\frac{1}{2}\cdot {f}_{\text{tot},N}^{{~}^{2}\!\left/ \!{~}_{3}\right.}\cdot \left[{l}_{N}+{\left(\frac{{f}_{\text{tot},N/O1/O2}}{{f}_{\text{tot},N}}\right)}^\frac{1}{3}\cdot {l}_{M/O1/O2}\right]}{D\cdot {A}_{N}}\cdot {V}_{N}\left(t\right)\ne {\tau }_\text{D}\left(t\right)$$

34 $${\tau }_{\text{c},N}^{\prime}\left(t\right)=\frac{1}{{f}_{\text{tot},N}\cdot {Q}_{N}\left(t\right)}\cdot {f}_{\text{tot},N}\cdot {V}_{N}\left(t\right)={\tau }_{\text{c},N}\left(t\right)$$

Since volume and volume flow are factorized in the same way, the convective time constants of the symmetrical and asymmetrical models are identical (Eq. 34). However, the factorization changes the diffusive time constants in the asymmetric model compared to the symmetric model (Eq. 33). Thus, upscaling of the geometry parameters leads to an increased ${\tau }_{\text{D},N}^{\prime}$, while downscaling lowers ${\tau }_{\text{D},N}^{\prime}$.

### Simulation and evaluation of the washout curves

#### Study design

**Fig. 4:**
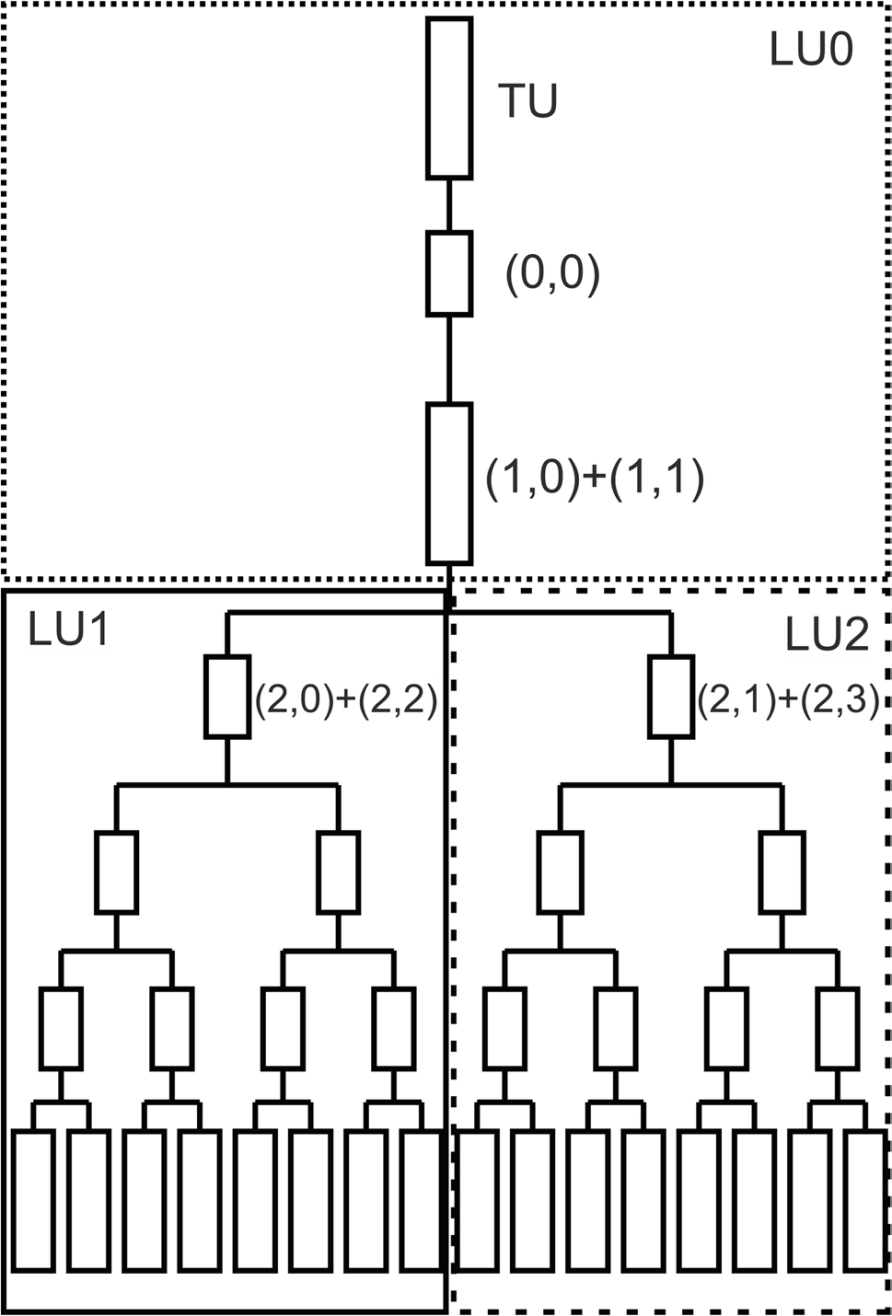
Schematic drawing of an asymmetric lung model where the asymmetry is in area 2. LU0 summarizes the TU as well as the AEs from areas 0 and 1. LU1 contains all AEs starting from the even parent AEs (2,0) and (2,2) and LU2 accordingly all from the odd parent AEs (2,1) and (2,3)

To observe the dependency of asymmetry on the distribution of He within one area, one of the areas 1 to 5 is designed asymmetrically. It is assumed that within an area *a*, all even AEs as well as odd AEs have the same fractional factor *f*(*a*,*e*) (Eq. 35). Based on this assumption, the model can be grouped into 3 lung units. LU0 summarizes all areas above the area where the asymmetry is applied. LU1 contains all AEs starting from the even parent AEs and LU2 accordingly all from the odd parent AEs, as can be seen in Fig. 4, where in area 2 the asymmetry has been applied. This approach is a modification of the method used by Paiva and Engel [46].

The factors *f*_c_ of 1.0, 1.25, 1.50, and 1.75 are chosen for the simulations, where 1 stands for a symmetrical model. This leads to volume ratios $\left(\frac{{V}_{{\mathrm{LU}}{1}}}{{V}_{{\mathrm{LU}}{2}}}\right)$ between LU1 and LU2 of $\left(\frac{1.25}{\mathrm{0.75}}\right)$, $\left(\frac{1.50}{\mathrm{0.50}}\right)$, and $\left(\frac{1.75}{\mathrm{0.25}}\right)$, respectively.

35 $$f\left(a,e\right)=\left\{\begin{array}{cc}{f}_{\text{c},}& e\in \left\{0, 2, 4.,\dots {2}^{a-1}\right\}\\ \begin{array}{c}2-{f}_{\text{c}},\\ 1,\end{array}& \begin{array}{c}e\in \left\{1, 3, 5,\dots {2}^{a-2}\right\}\\ \text{else}\end{array}\end{array}\right.$$

#### Initial and boundary conditions

At the beginning of the simulation (*t* = 0 s), pure nitrogen is present in the entire lung model. During inspiration, the He concentration at the model input is set to 100%, so that pure tracer gas is inhaled. During expiration, the He concentration at the model input is set to 0%. Due to this stepwise change in concentration at the input, incorrect concentration jumps occur in the washout curves, which are caused by the diffusional resistance at the input of the first AU. To ensure that this change in concentration at the model input does not lead to incorrect concentration jumps in the washout curves, the diffusional resistance of the first AU is not 1.33 $\frac{\mathrm{s}}{{\mathrm{cm}}^{3}}$ as in the other AUs of the TU, but 10^100^ $\frac{\mathrm{s}}{{\mathrm{cm}}^{3}}$ (Table 1).

#### Simulation and evaluation of the washout data

The simulation time is set to 200 s with a breathing period of *T* = 4 s (2 s inspiration, 2 s expiration), which corresponds to a respiratory rate of 15 1/min. In accordance to Henry et al. [44], the phase 3 slopes S_3_ are determined from the last 25% of the exhaled tidal volume by applying a trend analysis in MATLAB^®^.

In order to investigate the course of the diffusive interaction between LU1 and LU2 over the exhalation cycles (breath number *n*) and its influence on the phase 3 slope, the concentration differences $\Delta {\overline{\chi }}_\text{N2}\left({t}_{n}\right)$ at the time points *t*_*n*_ (Eq. 39, Eq. 37) are calculated from the mean nitrogen concentrations $\Delta {\overline{\chi }}_\text{N2,LUi}\left({t}_{n}\right)$ of the lung units LU1 and LU2 (Eq. 38). At these time points, the interaction between LU1 and LU2, taking into account the transit time $\Delta {\overline{\chi }}_{\text{LU}i}$ through LU0, which can be approximated by Eq. 36, causes the beginning of the phase 3 slope.

36 $$\Delta {t}_\text{LU0}=\frac{{V}_\text{LU0}}{250 \text{ ml/s}}$$

37 $${t}_{n}=3.5s-\Delta {t}_\text{LU0}+\left(n-1\right)\cdot T;1\le n\le 50$$

38 $$\Delta {\overline{\chi }}_{\text{N2,LU}i}\left({t}_{n}\right)=\frac{\sum\nolimits_{\forall j\in \text{LU}i}\chi_{\text{N2},i,j}\left({t}_{n}\right)\cdot {V}_{ij}\left({t}_{n}\right)}{\sum\nolimits_{\forall k\in \text{LU}i}{V}_{i,k}\left({t}_{n}\right)};i\in \left\{\mathrm{1,2}\right\}$$

39 $$\Delta {\overline{\chi }}_\text{N2}\left({t}_{n}\right)={\overline{\chi }}_{\text{N2,LU}1}\left({t}_{n}\right)-{\overline{\chi }}_{\text{N2,LU}2}\left({t}_{n}\right)$$

To analyze the dependence between the phase 3 slopes S_3_(*n*) and the mean N_2_ concentration differences $\Delta {\overline{\chi }}_{N2}\left({t}_{n}\right)=\Delta {\overline{\chi }}_{N2}\left({t}_{n}\right)$, the ratio f_S3_(*n*) between these two parameters is calculated (Eq. 40).

40 $${f}_\text{S3}\left(n\right)=\frac{{\text{S}}_{3}\left(n\right)}{\Delta {\overline{\chi }}_\text{N2}\left(n\right)}$$

The normalized phase 3 slopes were calculated from the ratio of S_3_(*n*) and the time-averaged N_2_ concentration of the *n*th expiratory cycle.

## Results

### Symmetrical model

#### Situation at the end of inspiration

**Fig. 5:**
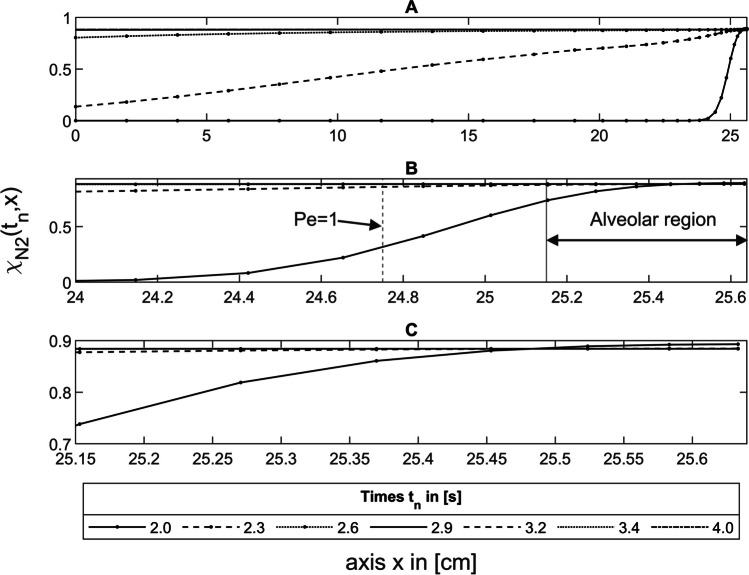
Nitrogen concentration curves in the symmetric model during expiration between 2.0 and 4.0 s for He-N_2_ along the entire lung axis *x* (**A**), at the transition (Pe = 1, vertical dashed lines) between the convection- and diffusion-dominated zone (**B**), and in the alveolar region (**C**)

Figure 5 shows the simulated N_2_ concentration distributions during the expiration of the first breath between 2.0 and 4.0 s for He-N_2_ along the entire lung axis *x* (A), at the transition between the convection- and diffusion-dominated zone (B), and in the alveolar region (G–I).

At the end of inspiration after 2.0 s, there is pure He in the anterior part of the dead space (Fig. 5A); the end of which is marked with a vertical solid line in Fig. 5B. The nitrogen concentration then rises in an s-shape to the end of the alveolar region, which corresponds to the area where the inhaled He has mixed with the N_2_.

#### Expiration

**Fig. 6:**
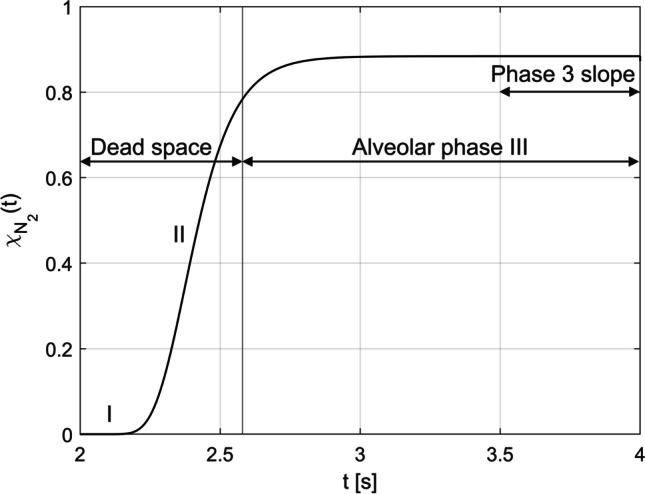
Simulated N_2_ washout curve of the first breath for He-N_2_, divided into three phases for the dead space (I, II) and the alveolar phase (III)

The expiration is divided into three phases, as shown in Fig. 6. In phase I, the He is exhaled from the dead space, which has not mixed with the nitrogen contained in the model. In phase II, the nitrogen concentration increases as the He has mixed with the N_2_ during inspiration (Fig. 5B). In the third phase (III), which begins 0.6 s after the start of expiration (Fig. 6), taking into account the transit time $\Delta {t}_\text{Dead}$ (Eq. 27), the gas mixture coming from the alveoli is expired. During this phase, the nitrogen concentration of the exhaled He-N_2_ reaches a plateau (Fig. 6) caused by a homogeneous concentration distribution in the alveolar region (Fig. 5C), which must have occurred there between 2.0 s (2.6 s-$\Delta {t}_\text{Dead}$) and 3.4 s (4.0 s-$\Delta {t}_\text{Dead}$) considering $\Delta {t}_\text{Dead}$. This plateau means a S_3_ of 0.

### Washout of He-N2 in the asymmetric model

#### Situation at the end of inspiration

**Fig. 7:**
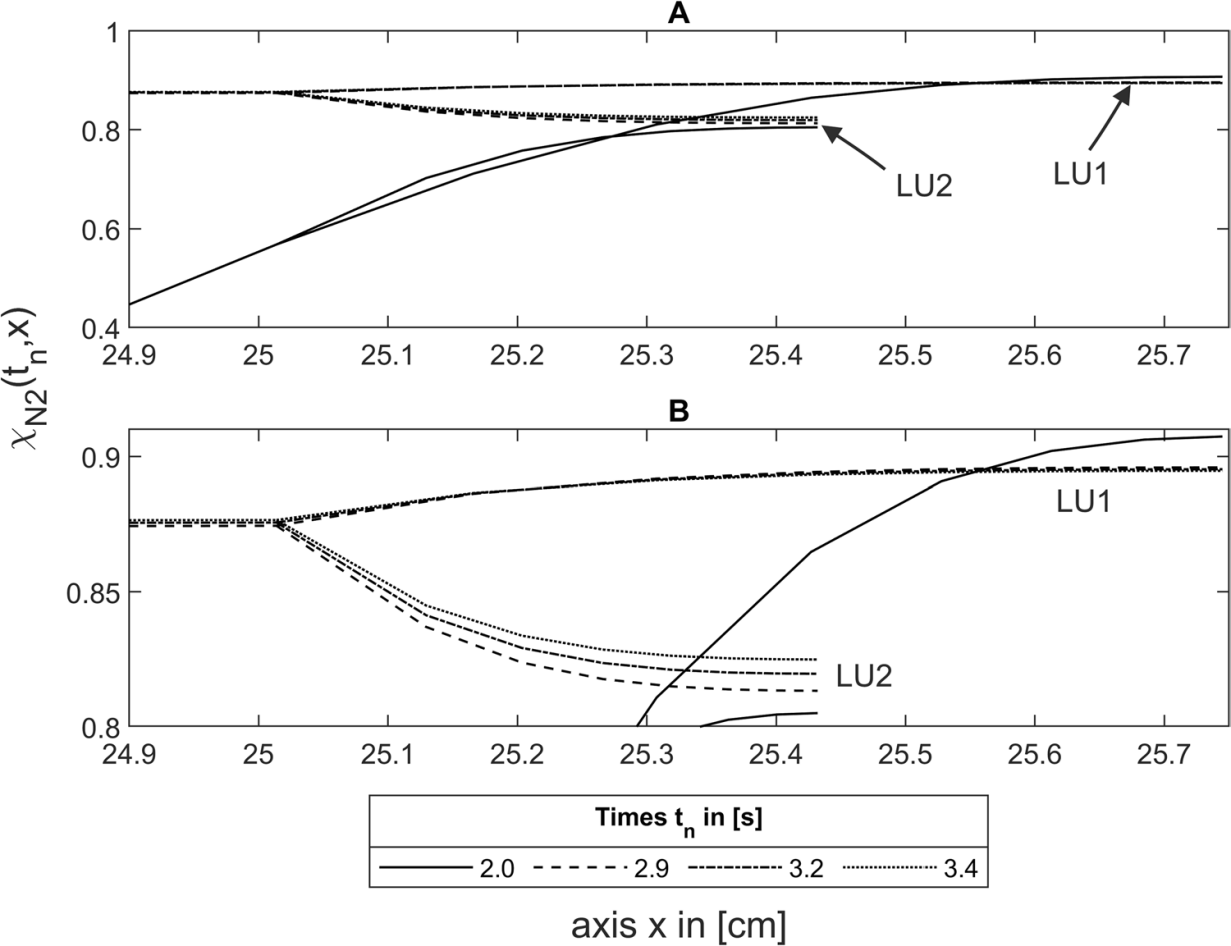
Nitrogen concentration in the asymmetric model during expiration at 2.0 s, 2.9 s, 3.2 s, and 3.4 s along the lung axis *x* for He-N_2_, where the asymmetry with a volume ratio of $\frac{{1}\mathrm{.}{75}}{{0}\mathrm{.}{25}}$ is in the area 4. **A** and **B** represent the same section of the lung axis, but with different time scales. See text for details

At the end of inspiration after 2.0 s, under asymmetric conditions, a concentration gradient forms between the larger lung unit LU1 and the smaller LU2 (Fig. 7A, B) because more tracer gas diffuses into LU2 than into LU1 during inspiration due to their smaller diffusion time constants ${\tau }_{\text{D},N}^{\prime}$ (Eq. 33). Consequently, the nitrogen concentration in LU2 is lower than in LU1.

#### Cause of phase 3 slopes

Due to the concentration gradient between LU1 and LU2, N_2_ diffuses from the larger LU1 via the bifurcation into the smaller LU2, resulting in an increasing nitrogen concentration in this unit during expiration (Fig. 7B). However, due to this nitrogen accumulation in LU2, the concentration gradient between LU1 and LU2 decreases and thus the diffusive transport between these two units. As a result, more N_2_ is transported from the bifurcation to the TU, leading to an increasing N_2_ concentration in the washout curve, which causes the phase 3 slope. The phase 3 slope of the first breath measured between 3.5 s and 4.0 s thus results from this diffusive interaction (Fig. 7B) between 2.9 s (3.5 s-$\Delta {t}_\text{LU0}$) and 3.4 s (4.0 s-$\Delta {t}_\text{LU0}$), taking into account the transit time $\Delta {t}_\text{LU0}$ (Eq. 36), which corresponds to $\Delta {t}_\text{Dead}$ for the example shown in Fig. 7, where the asymmetry is in the area 4.

**Fig. 8:**
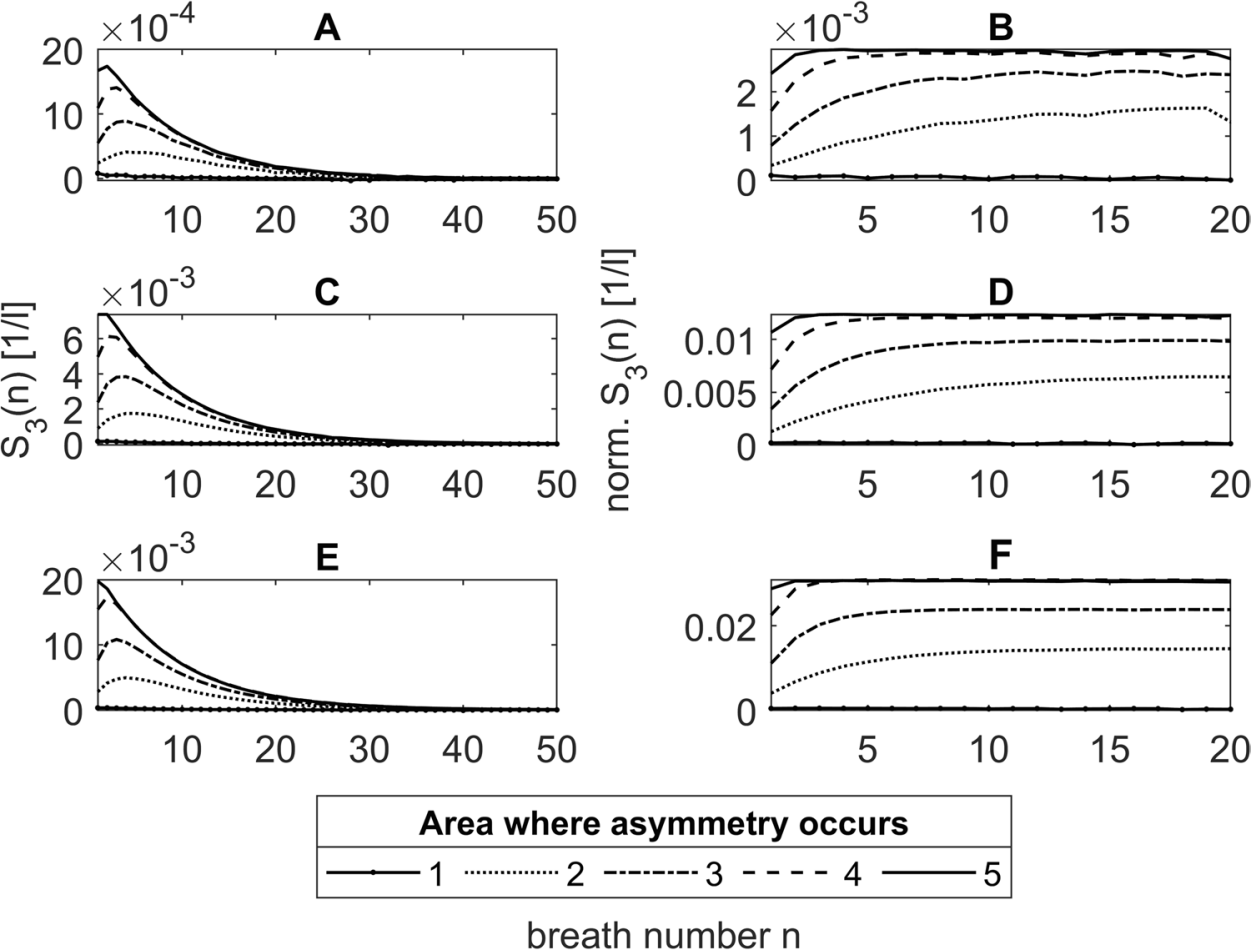
S_3_(*n*) (**A**, **C**, **E**) and normalized (norm.) S_3_(*n*) (**B**, **D**, **F**) of the He-N_2_ washout curves under asymmetric conditions for the volume ratios between LU1 and LU2 of $\frac{1.25}{0.75}$ (**A**, **B**), $\frac{1.50}{0.50}$ (**C**, **D**), and $\frac{1.75}{0.25}$ (**E**, **F**), with asymmetries in areas 1 to 5. Norm. S_3_(*n*) is shown only for the first 20 breaths, since the calculation errors for small S_3_(*n*) are too large

Figure 8 (A, C, E) shows the phase 3 slopes of the He-N2 washout curves under asymmetric conditions for the volume ratios between LU1 and LU2 of $\frac{1.25}{\mathrm{0.75}}$, $\frac{1.50}{\mathrm{0.50}}$ and $\frac{1.75}{\mathrm{0.25}}$ as a function of breath number *n*, with asymmetries between LU1 and LU2 in areas 1 to 5. An asymmetry in the convection-dominated zone in area 1 does not lead to a phase 3 slope (Fig. 8 A, C, E solid lines with dots) since Pe at the entrance of that area is much greater than 1 (Table 1) and therefore gas transport between LU1 and LU2 by diffusion is negligible.

#### Change in phase 3 slopes over *n*

**Fig. 9:**
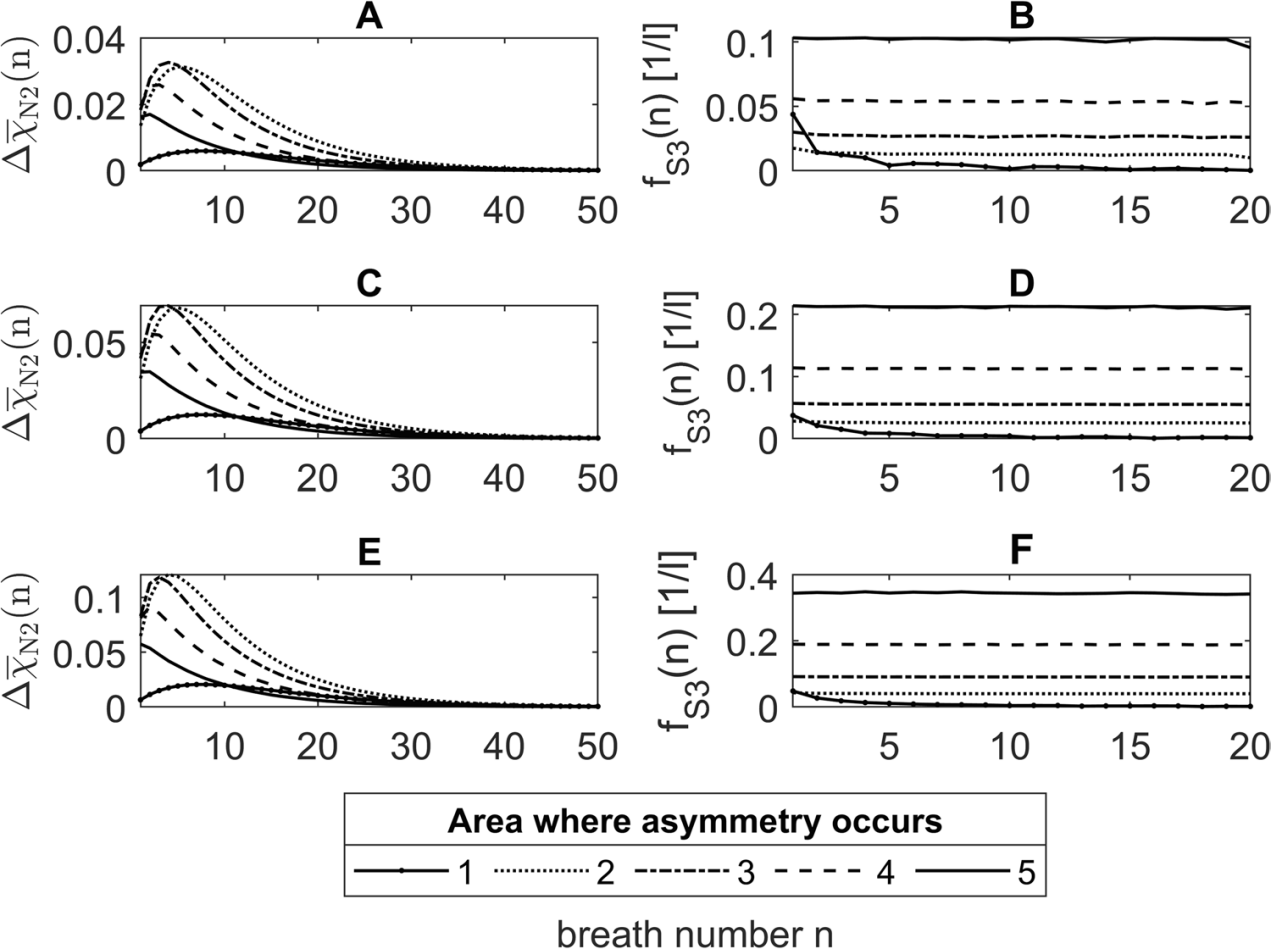
$\Delta {\overline{\chi }}_\text{N2}\left(n\right)$ and *f*_S3_(*n*) of the He-N_2_ washout under asymmetric conditions for the volume ratios between LU1 and LU2 of $\frac{{1}\mathrm{.}{25}}{{0}\mathrm{.}{75}}$ (**A**, **B**), $\frac{{1}\mathrm{.}{50}}{{0}\mathrm{.}{50}}$ (**C**, **D**), and $\frac{{1}\mathrm{.}{75}}{{0}\mathrm{.}{25}}$ (**E**, **F**) where the asymmetry occurs in areas 1 to 5. *f*_S3_(*n*) is shown only for the first 20 breaths, since the calculation errors for small $\Delta {\overline{\chi }}_\text{N2}\left(n\right)$ and S_3_(*n*) are too large

The functions for S_3_(*n*) in Fig. 8(A, C, E) show, with one exception (Fig. 8E solid line), characteristic curves for asymmetries in the areas 2 to 5. First, the S_3_(*n*) rises with increasing *n* until a maximum is reached. Then, S_3_(*n*) decreases exponentially. The reason for this is the concentration gradient between LU1 and LU2, which not only changes in the course of one breath, but also depends on the breath number *n*. That can be explained by the mean concentration differences $\Delta {\overline{\chi }}_\text{N2}\left(n\right)$ (Eq. 39) between LU1 and LU2 (Fig. 9A, C, E), which are proportional to S_3_(*n*), since the ratio *f*_S3_(*n*) between both is constant as can be seen in Figure (Fig. 9B, D, F). The increase in $\Delta {\overline{\chi }}_\text{N2}\left(n\right)$ on the first breaths is due to the smaller diffusive time constants ${\tau }_{\text{D}}^{\prime}$ in LU2, which leads to a faster N_2_ washout in this lung unit compared to LU1. Caused by the resulting accumulation of He, however, less freshly inhaled helium diffuses into LU2 during inspiration. Consequently, $\Delta {\overline{\chi }}_\text{N2}\left(n\right)$ and thus S_3_(*n*) decrease.

**Fig. 10:**
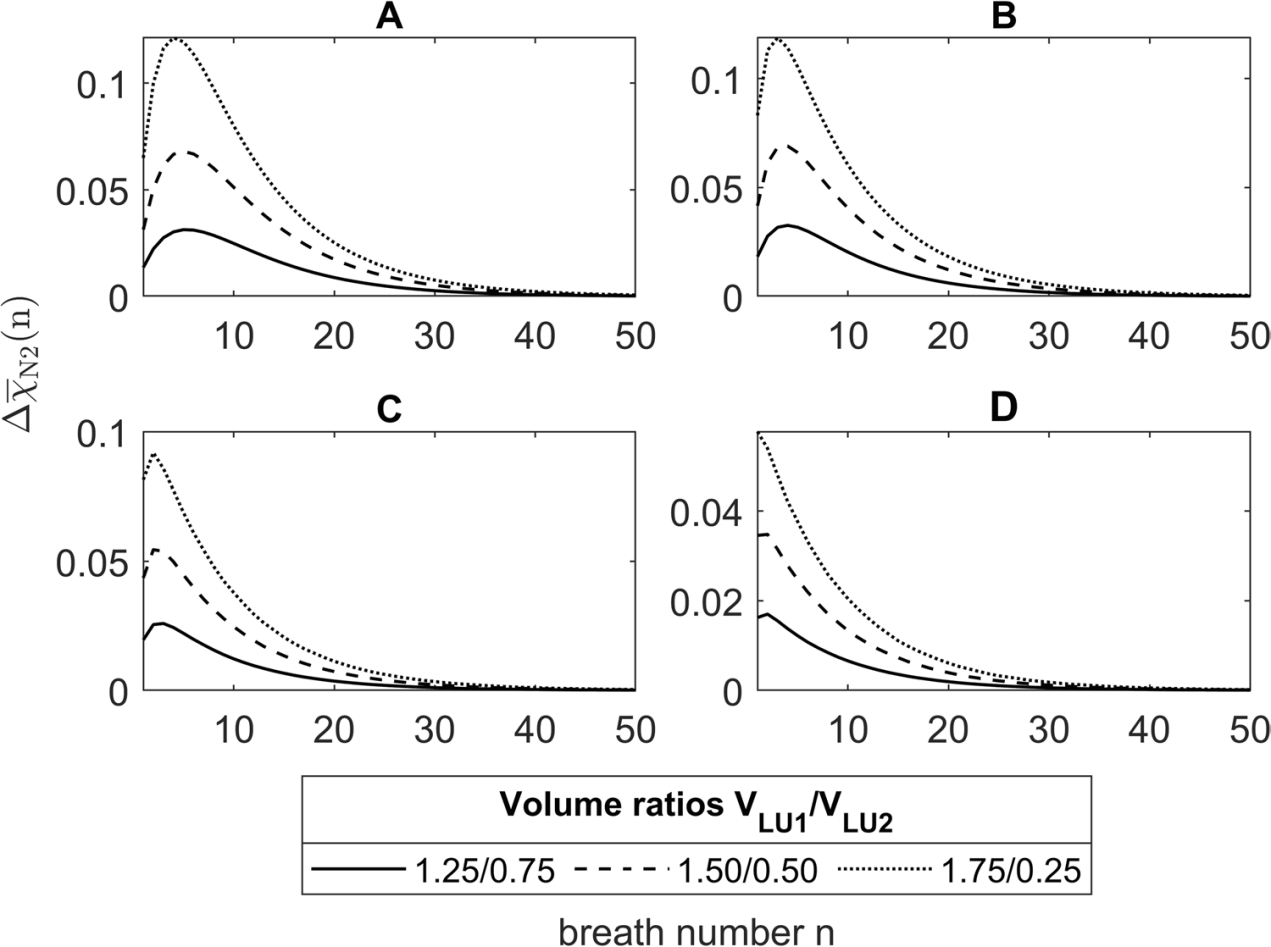
$\Delta {\overline{\chi }}_\text{N2}\left(n\right)$ of the He-N_2_ washout under asymmetric conditions for the volume ratios between LU1 and LU2 of $\frac{{1}\mathrm{.}{25}}{{0}\mathrm{.}{75}}$ (solid lines), $\frac{{1}\mathrm{.}{50}}{{0}\mathrm{.}{50}}$ (dashed lines), and $\frac{{1}\mathrm{.}{75}}{{0}\mathrm{.}{25}}$ (dotted lines) where the asymmetry occurs in areas 2 to 5 (A to D)

**Fig. 11:**
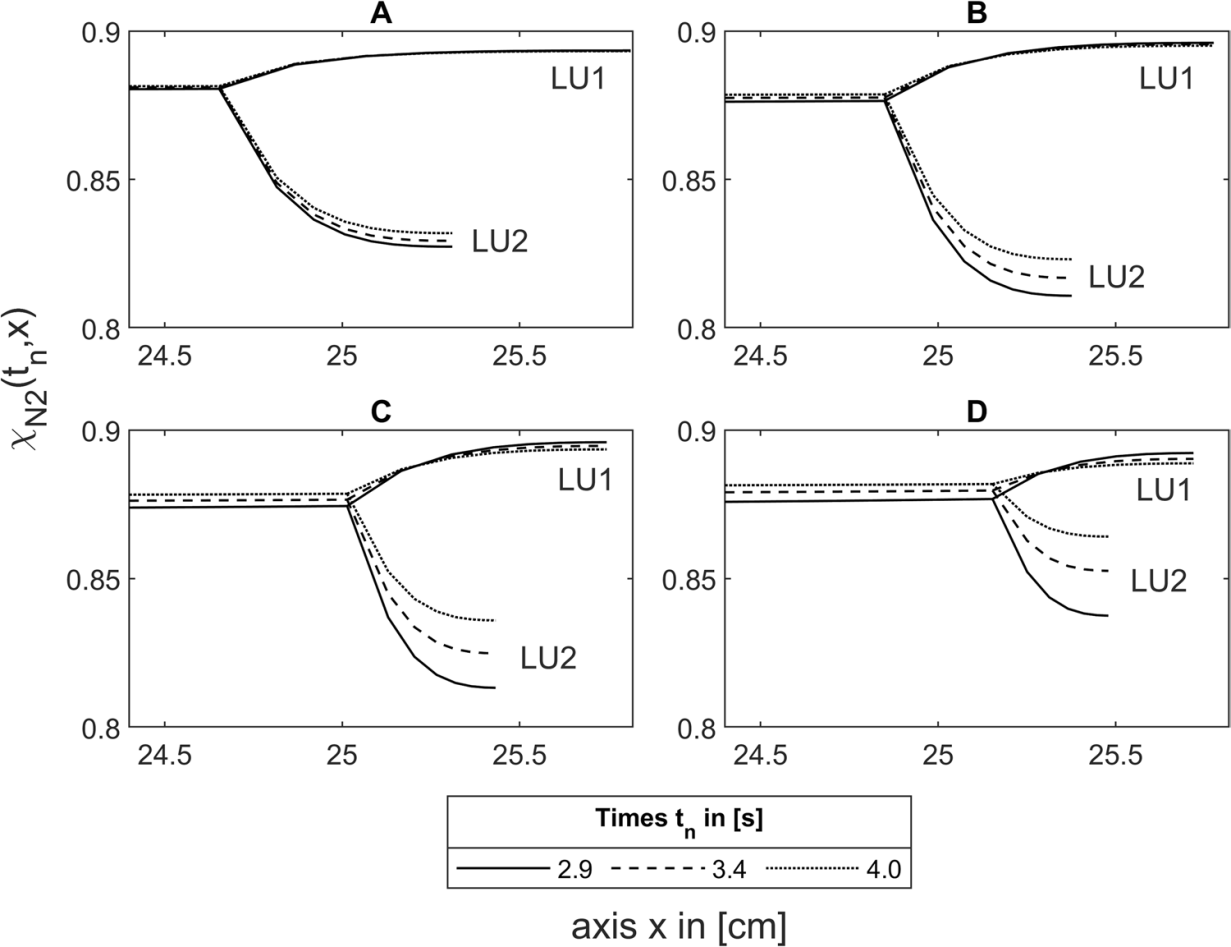
Nitrogen concentrations in the asymmetric model during expiration at 2.9 s, 3.4 s, and 4.0 s along the lung axis *x* for He-N_2_ in LU1 and LU2, with asymmetries of $\frac{1.75}{\mathrm{0.25}}$ are in the area 2 (**A**), 3 (**B**), 4 (**C**), and 5 (**D**)

However, with a large volume ratio of $\frac{1.75}{\mathrm{0.25}}$, where the asymmetry is in the area 5, the maximum of S_3_(*n*) and $\Delta {\overline{\chi }}_\text{N2}\left(n\right)$ is at the first breath (Fig. 8E solid line). To explain this, Fig. 10 shows the concentration differences $\Delta {\overline{\chi }}_\text{N2}\left(n\right)$ from Fig. 9 for the volume ratios $\frac{1.25}{\mathrm{0.75}}$ (solid lines), $\frac{1.50}{\mathrm{0.50}}$ (dashed lines), and $\frac{1.75}{\mathrm{0.25}}$ (dotted lines) with asymmetries between LU1 and LU2 in the areas 2 to 5 (A to D). It can be seen that the maxima of $\Delta {\overline{\chi }}_\text{N2}\left(n\right)$ in image parts A to D shift from area 2 (A) to the lung periphery in area 5 (D) towards the first breath. As the diffusive transport in the lung periphery becomes stronger and the volumes and lengths of LU1 and LU2 become smaller, the smaller lung unit LU2 accumulates more with N_2_ during expiration, whereas the N_2_ concentration in LU1 decreases (Fig. 11A–D). As a result, the N_2_ washout in LU2 is progressively slowed down while it is accelerated in LU1. Due to this increasing diffusive interaction between LU1 and LU2, the maximum concentration difference of $\Delta {\overline{\chi }}_\text{N2}\left(n\right)$ between these two lung units shifts in the direction of the first breath.

#### Normalized phase 3 slopes

Figure 8 (B, D, F) shows the normalized phase 3 slopes of He-N_2_. For asymmetries in the areas 2 to 5, these correspond approximately to monoexponential growth functions. For large *n*, the calculation error increases, so that the normalized S_3_(*n*) becomes unstable. For this reason, only the first 20 breaths are shown (Fig. 8B, D, F). The steady-state value indicates that the exponential decreases in the mean nitrogen concentration of the washout curves and the concentration difference $\Delta {\overline{\chi }}_\text{N2}\left(n\right)$ underlying the phase 3 slope S_3_(*n*) are identical. The increase until the steady state value is reached results from the different N_2_ washouts of LU1 and LU2 (see above).

## Discussion

### Normalized phase 3 slope

The results presented here show that in the symmetric model with He as tracer gas, a homogeneous concentration occurs in the alveolar space during expiration, so that no phase 3 slope is generated (Section 3.1.2). However, under asymmetric conditions, a diffusive interaction between adjacent lung units occurs during expiration, with nitrogen diffusing from the larger lung unit LU1 to the smaller unit LU2, leading to a phase 3 slope (Section 3.2.2). Due to the ratio of the phase 3 slope, which is proportional to the concentration difference (Section 3.2.3), and the mean nitrogen concentration, the normalized phase 3 slope increases during the first breaths before reaching a plateau (Fig. 8).

This typical curve shape is consistent with the results of clinical studies [47] and model simulations [24], although only a qualitative comparison of the curves is possible at this point, as the exact course depends on the complexity of the airway network, as described in Section 4.2. For the purpose of this paper, however, a qualitative comparison is sufficient to show that the underlying diffusive interaction of parallel connected lung units, which causes the so-called Pendelluft [47], can be simulated with this model.

Abbasi and Bozorgmehry Boozarjomehry [24] were also able to simulate this typical curve shape of the normalized phase 3 slopes with their model using symmetrical specific ventilation of the lung units (without CDI) and N_2_ as tracer gas. Their model, based on a modified Hagen-Poiseuille equation to calculate the volume flow and an extended convection-diffusion equation to calculate the concentration distribution, was implemented in C++ and solved numerically. However, this “classical” implementation can be very complicated, as the transport equations of the individual nodes have to be combined by the software developer into a system of differential equations, which makes it difficult to understand the interactions of the computational nodes, especially in large and complex airway networks. On the other hand, the model presented here offers the great advantage and the novelty that, due to its modular structure, any airway network can be realized by placing and wiring elementary computational nodes based on electrical components (Fig. 3). This “wiring” of the computational nodes makes it comparatively easy to realize even larger and more complex airway networks and to monitor and understand the interaction of the computing nodes with each other.

### Model complexity

The breath number at which the maximum of the normalized phase 3 slopes is reached depends on the volume ratio and the position of the asymmetry (Fig. 8). The more peripheral the asymmetry of the transition between the convection- and diffusion-dominated zone (Table 1), the sooner the plateau is reached, since the increasing diffusive interaction combined with the decreasing volumes of LU1 and LU2 promotes a faster equalization of concentration between LU1 and LU2 (Section 3.2.3). This observation can be made since a simple, symmetrically branched model geometry is used to reproduce the DCDI, in which anatomical asymmetries have been inserted at certain points, so that diffusive interactions only occur between two adjacent lung units.

However, in models with more complex asymmetric branching geometries like that of Abbasi and Bozorgmehry Boozarjomehry [24], the normalized phase 3 slopes are affected both by diffusive interactions within the airway networks of the diffusion-dominated zone and between them at the level of the transition between the convection- and diffusion-dominated zones. Due to this interaction, the normalized phase 3 slopes caused by the DCDI reach their plateau after approximately 6 breaths [24].

Since this interaction determines the MBW parameters, the models must have a sufficiently large anatomical complexity to optimally reproduce the gas distribution in the lungs, which can potentially be realized by the approach presented here (Section 4.1). However, numerical models have the disadvantage that the computation time increases with increasing complexity, whereas the approach presented here has the advantage that the electrical components can in principle be constructed as hardware devices, so that the simulation can be performed in real-time regardless of the complexity of the model. For instance, the simulation of a washout process with a simulation time of 200 s (Section 2.5.3) would also require exactly 200 s. The recording of washout curves (Fig. 6), the analysis of the diffusive interaction between parallel lung units (Fig. 7, Fig. 11), and the evaluation of output parameters such as the phase 3 slope (Fig. 8) could be performed in real time during the simulation as the electrical voltages and currents could be measured and processed directly. For this reason, such a hardware simulator could be used in clinical studies for efficient and rapid data analysis.

In addition, the use of electrical components makes it possible to describe the anatomy of the airways as well as the transport processes convection and diffusion by simple electrical resistances and capacitances, thus reducing the complexity of the interaction between these parameters. This leads to the concept of convective and diffusive time constants (Eqs. 31, 32), which are the product of capacitances and resistances, and can be used to interpret the data obtained (Section 3.2.1). This concept allows, for instance, a clear and simple explanation of the origin of the concentration difference between LU1 and LU2 at the end of the inspiration (Section 3.2.1), which causes the phase 3 slope (Section 3.2.2).

### Lung mechanics

Verbanck and Foy [23] showed in their study that the gas distribution and thus the phase 3 slope is also influenced by lung units that have different mechanical properties. In particular, the distribution of the volume flows in the airway generations 5 to 7 (convection-dominated area) seems to be of decisive importance for the CDI. The authors used a combination of a ventilation model and an MBW model to simulate the lung mechanics [48, 49]. The ventilation model calculates the volume flow or velocity distribution, which is used in the MBW model to simulate the concentration distribution. As the authors focused on the analysis of the CDI in their study, diffusive gas transport was not considered and the acini were modelled as elastic spheres. Both models were implemented in MATLAB^®^ and solved numerically [49].

The model presented here is not only an extension of the MBW model of Verbanck and Foy [23], in which both convective and diffusive gas transport are taken into account, but also offers an alternative way of implementing the underlying system of equations on the basis of electrical components, the advantages of which have already been explained in Sections 4.1 and 4.2. In contrast to Verbanck and Foy [23], the model presented in this paper does not use a separate ventilation model to calculate the volume flows and therefore does not consider the CDI, as rectangular functions (Section 2.4.2) are used for the first model test to prove that the DCDI could be modelled. Therefore, a volume asymmetry in area 1, starting from airway generation 5 (Fig. 1), does not lead to a phase 3 slope with the model presented in this paper, since the specific ventilations of LU1 and LU2 are identical, i.e., volume and volume flow are factorized in the same way (Section 2.4.4), resulting in convective homogeneous ventilation. This condition was chosen to test whether the model can reproduce the DCDI. Since area 1 is located in the convection-dominated zone, the diffusive interaction between LU1 and LU2 is negligible (Section 3.2.2). In future work, the model presented here will be extended to allow heterogeneous ventilation of lung units connected in parallel and thus to simulate and analyze both the CDI and the DCDI. In order not to diminish the advantages of using electrical components mentioned in Sections 4.1 and 4.2, the ventilation model of [48] is unsuitable. However, lung mechanics can also be modelled using an electrical equivalent circuit in which the electrical variables capacitance, resistance, current, and voltage correspond to the parameters compliance, flow resistance, volume flow, and pressure [36]. A network of these electrical components could be used in parallel with the model presented here, which determines the gas concentration, to calculate the volume flows.

### Alternative to the phase 3 slope

In their study, Bates et al. [30] compared the results of the “classical” method for analyzing ventilation inhomogeneities, which investigated the normalized phase 3 slopes of the washout curves, with the results of a method in which the washout curves were adjusted with 4 degrees of freedom. The latter method is based on the model concept, in which the lung consists of several parallel alveolar units, each supplied by its own airways and converging at the model entrance [30]. The authors found that the phase 3 slopes are very sensitive to irregularities in patient respiration and the distinction between the end of the second and the beginning of the third phase of the washout curves. In contrast, the parameters from the curve-fitting method were less sensitive to irregular breathing patterns.

Phase 3 slopes were calculated in this paper to analyze the diffusive interaction of adjacent lung units (Section 3.2) to demonstrate the feasibility of the new model approach. For this purpose, the breathing patterns are initially uniform (Section 2.4.2) so that normalized phase 3 slopes can be determined under ideal conditions. However, the approach presented here is not limited to uniform respiration, but can be extended for any volume flow (Section 4.3).

The disadvantage of the model presented by Bates et al. [30] is that a lung consisting of only parallel alveolar units does not adequately represent the complex interactions in a branched airway network. In a branched airway network, as used in this work (Fig. 1), the distribution of tracer gas in the airways can be simulated and visualized (Figs. 5, 7, 11), so that the diffusive and convective interactions in the airways can be analyzed.

Further work is needed to analyze the difficulties in determining the phase 3 slopes, to determine alternative less sensitive parameters from the washout curves, and to prove model approaches such as that of Bates et al. [30].

### Assumptions and boundary conditions

The Taylor dispersion, which is caused by large velocity gradients [40], was not taken into account as it could distort the analysis of the correlation between the phase 3 slope and the diffusive interaction between the neighboring lung units LU1 and LU2. This is particularly the case in the convection-dominated zone where the Taylor dispersion leads to increased mixing between the nitrogen and the inhaled helium and could therefore affect the phase 3 slope. In order to investigate this mechanism and its effects on the phase 3 slope in later work, the diffusion coefficient in Eq. 29 and Eq. 30 can be replaced by the dispersion coefficient.

For the studies performed in this paper, pure helium is inhaled (Section 2.5.2), which does not correspond to the clinical conditions [47] and is non-physiological. However, this deviation from clinical protocols is a common method [44, 50] and crucial for system analysis to investigate the influence of different tracer gases such as He, O_2_, and SF_6_ (Section 4.6) on the washout curves and to compare the results with each other. The concentrations can be modified for later clinical applications.

The model input contains both the measuring point for recording the washout curves and the gas supply, which switches the tracer gas on and off according to the breathing phase (Section 2.5.2). In order to avoid incorrect jumps in the washout curves which can occur due to this change in concentration, the value of the diffusion resistance at the model input has been set to a value of 10^100^ $\frac{\mathrm{s}}{{\mathrm{cm}}^{3}}$ for the sake of simplicity (Table 1). Otherwise, as is common in clinical devices [51], an additional dead space volume would have to be placed between the gas supply and the measurement point.

### Outlook

In further work, it must be investigated whether the model can reproduce washouts with other test gases such as SF_6_ and O_2_. A mechanical setup is currently being worked on with which the results of the numerical model can be compared with those from in vitro models.

In addition, it must be clarified whether a hardware setup with such electrical components can be realized. This does not only include simple model geometries as in the model presented here, but also for more complex, branched airway networks. Such a hardware-based setup could be used to create a patient-specific model, which would be useful as a diagnostic tool for the assessment of the small airways and therefore for the early detection of obstructive pulmonary diseases.

## Conclusion

We present a new numerical lung model that describes the anatomical and physiological properties of the lung, as well as convection and diffusion, with electrical components to simulate inert gas washout methods. Our results show that we can simulate the characteristic shape of the normalized phase 3 slopes caused by the DCDI with the model presented here. The shape depends on the volume ratio of parallel lung units and the area where the asymmetry occurs. DCDI occur in the transition and in the diffusion-dominated zone, whereas in the convection zone, a diffusive interaction between parallel lung units is negligible. Therefore, the use of electrical components that model the gas transport within the lung could potentially form the basis of a hardware-based simulator for real-time computation and data analysis during patient diagnosis.
